# PLAZA 5.0: extending the scope and power of comparative and functional genomics in plants

**DOI:** 10.1093/nar/gkab1024

**Published:** 2021-11-08

**Authors:** Michiel Van Bel, Francesca Silvestri, Eric M Weitz, Lukasz Kreft, Alexander Botzki, Frederik Coppens, Klaas Vandepoele

**Affiliations:** Department of Plant Biotechnology and Bioinformatics, Ghent University, Technologiepark 71, 9052 Ghent, Belgium; VIB Center for Plant Systems Biology, Technologiepark 71, 9052 Ghent, Belgium; Department of Plant Biotechnology and Bioinformatics, Ghent University, Technologiepark 71, 9052 Ghent, Belgium; VIB Center for Plant Systems Biology, Technologiepark 71, 9052 Ghent, Belgium; Data Sciences Platform, Broad Institute of MIT and Harvard, Cambridge, MA 02142, USA; Institute of Biochemistry and Biophysics, Polish Academy of Sciences, Pawińskiego 5A 02-106 Warsaw, Poland; VIB Bioinformatics Core, 9052 Ghent, Belgium; Department of Plant Biotechnology and Bioinformatics, Ghent University, Technologiepark 71, 9052 Ghent, Belgium; VIB Center for Plant Systems Biology, Technologiepark 71, 9052 Ghent, Belgium; Department of Plant Biotechnology and Bioinformatics, Ghent University, Technologiepark 71, 9052 Ghent, Belgium; VIB Center for Plant Systems Biology, Technologiepark 71, 9052 Ghent, Belgium; Bioinformatics Institute Ghent, Ghent University, Technologiepark 71, 9052 Ghent, Belgium

## Abstract

PLAZA is a platform for comparative, evolutionary, and functional plant genomics. It makes a broad set of genomes, data types and analysis tools available to researchers through a user-friendly website, an API, and bulk downloads. In this latest release of the PLAZA platform, we are integrating a record number of 134 high-quality plant genomes, split up over two instances: PLAZA Dicots 5.0 and PLAZA Monocots 5.0. This number of genomes corresponds with a massive expansion in the number of available species when compared to PLAZA 4.0, which offered access to 71 species, a 89% overall increase. The PLAZA 5.0 release contains information for 5 882 730 genes, and offers pre-computed gene families and phylogenetic trees for 5 274 684 protein-coding genes. This latest release also comes with a set of new and updated features: a new BED import functionality for the workbench, improved interactive visualizations for functional enrichments and genome-wide mapping of gene sets, and a fully redesigned and extended API. Taken together, this new version offers extended support for plant biologists working on different families within the green plant lineage and provides an efficient and versatile toolbox for plant genomics. All PLAZA releases are accessible from the portal website: https://bioinformatics.psb.ugent.be/plaza/.

## INTRODUCTION

Since the initial release of the platform in 2009 (1), PLAZA has integrated genome annotations from a large variety of international data providers. The slow gradual increase in the number of available high-quality plant genomes, as exemplified by comparing the number of species in PLAZA 1.0 (9 species) (1) to PLAZA 2.5 (25 species) (2), has now grown into a veritable avalanche of new plant genomes (3). This acceleration can largely be attributed to the application of long-read sequencing and better scaffolding methods (4). The challenges posed by the associated increase in data volume are addressed in different ways by data integration platforms (5–7). The use of efficient and scalable workflows to perform comparative sequence analysis is required, as well as highly performant interactive visualizations to efficiently explore different data types (e.g. phylogenetic trees, gene colinearity between two or more genomes, complex gene orthology) for all or a selection of species (8).

The PLAZA platform offers an extensive online toolbox for plant and computational scientists to perform gene-based data analysis, explore and extract sequence and functional information, and generate evolutionary insights for the plant genomes integrated in the available PLAZA instances. To better understand the large phenotypic diversity encoded by different plant genomes, many different types of data are used: structural and functional annotations, gene families grouping homologous genes, phylogenetic trees assisting in the identification of orthologs and paralogs, and detailed information about genome organization (e.g. local gene colinearity or whole-genome synteny). Access to sequenced genomes for species of different plant families offers information about genes or pathways responsible for specific traits in cereals (9), fruit crops (10), legumes (11), trees (12), ornamental crops (13) or parasitic plants (14). Furthermore, comparative gene and genome analysis reveals how small- and large-scale gene duplications, as well as gene loss and transposons, are driving plant genome evolution and adaptation (15,16). Finally, genome sequencing and comparative analysis of basal or early-diverging plants provide new insights into the emergence and evolution of signaling pathways controlling the responses to diverse environmental conditions and the development of specific plant organs or cell types (17).

We initially designed the PLAZA platform to make comparative and functional genomics data analyses in plants possible through a user-friendly web interface, but over time the PLAZA platform also grew to serve as a data warehouse containing high-quality, curated genome and functional annotations, as well as gene family and gene orthology information. Besides bulk downloads, PLAZA also provides access to multiple data types through a RESTful Application Programming Interface (API), resulting in enhanced compatibility with workflow systems. Next to organizing (virtual) international workshops and training sessions, we provide extensive documentation and tutorials, elaborating on the data content, tools and third-party software that is used in the PLAZA build procedure.

Here, we present PLAZA 5.0 (available at https://bioinformatics.psb.ugent.be/plaza), the most recent iteration of the PLAZA platform for comparative, evolutionary, and functional genomics. This new release offers a two-fold increase in the number of available plant species, together with a better sampling of basal plants that serve as outgroups to the angiosperms. As in the previous two releases of the PLAZA platform, we created two instances, focusing either on monocotyledonous or dicotyledonous plants. The continuous development of PLAZA, with a strong focus on enhancing the user experience through easy to use yet powerful analysis tools and visualizations, allows for efficient exploration of new genomes and data types, enabling researchers to utilize ever increasing volumes of data.

## SPECIES AND DATA CURATION

### Species selection

We performed an extensive evaluation of publicly available plant genomes in order to determine which ones to include in the new PLAZA 5.0 instances. The inclusion of organisms for which the annotation exhibits insufficient quality, e.g. due to unassembled genome sequences or incomplete gene prediction, may lead to downstream errors such as the erroneous inference of presence/absence variation in gene families or incorrect collinearity detection (18).

For PLAZA 5.0 we used plaBiPD (https://www.plabipd.de) as the main resource to start the species selection. We extracted assembly statistics, such as genome size and N50 value, from over 400 published plant genomes. We used this information, which we deemed a proxy for genome completeness and contiguity, as a primary filter. Additional selection criteria were whether a species is an important model species (e.g. *Arabidopsis thaliana, Oryza sativa*), whether the species is of a high economic value (e.g. wheat), and whether the species has an evolutionary important place within the tree of life (e.g. outgroup species or species being the first representative of a specific plant clade). As a final criterion, we tried to cover as many plant families as possible, enhancing the power of evolutionary analyses as much as possible.

We selected a total of 134 species to be integrated in PLAZA 5.0 (see Supplementary Table S1), spread over two different instances: PLAZA Monocots 5.0 (53 species) and PLAZA Dicots 5.0 (100 species). This is a significant increase compared to their counterparts in PLAZA 4.0 (19), with 29 and 55 species, respectively. This near doubling in number of high-quality genomes is a clear indication of the application of next-generation long-read sequencing and improved scaffolding methods to characterize plant genomes (20).

A small group of reference species was selected to be present in both PLAZA 5.0 instances, in order to facilitate comparative and evolutionary genomics: five model species within the Dicot clade (*Arabidopsis thaliana*, *Glycine max*, *Populus trichocarpa*, *Solanum lycopersicum, and Vitis vinifera*), three species within the Monocot clade (*O. sativa*, *Zea mays* and *Vanilla planifolia*), one species each for the magnoliids and Ceratophyllales (*Magnolia biondii* and *Ceratophyllum demersum*), and a group of 10 basal plant species that act as outgroup to both the Dicots and Monocots (*Amborella trichopoda*, *Sequoiadendron giganteum*, *Selaginella moellendorffii*, *Anthoceros agrestis*, *Marchantia polymorpha*, *Physcomitrium patens*, *Chara braunii*, *Prasinoderma coloniale*, *Chlamydomonas reinhardtii* and *Micromonas commoda*). The reference species were selected based on their status as model species, as well as phylogenetic coverage (e.g. *Vanilla planifolia* was selected as non-Poales representative).

**Figure 1.**
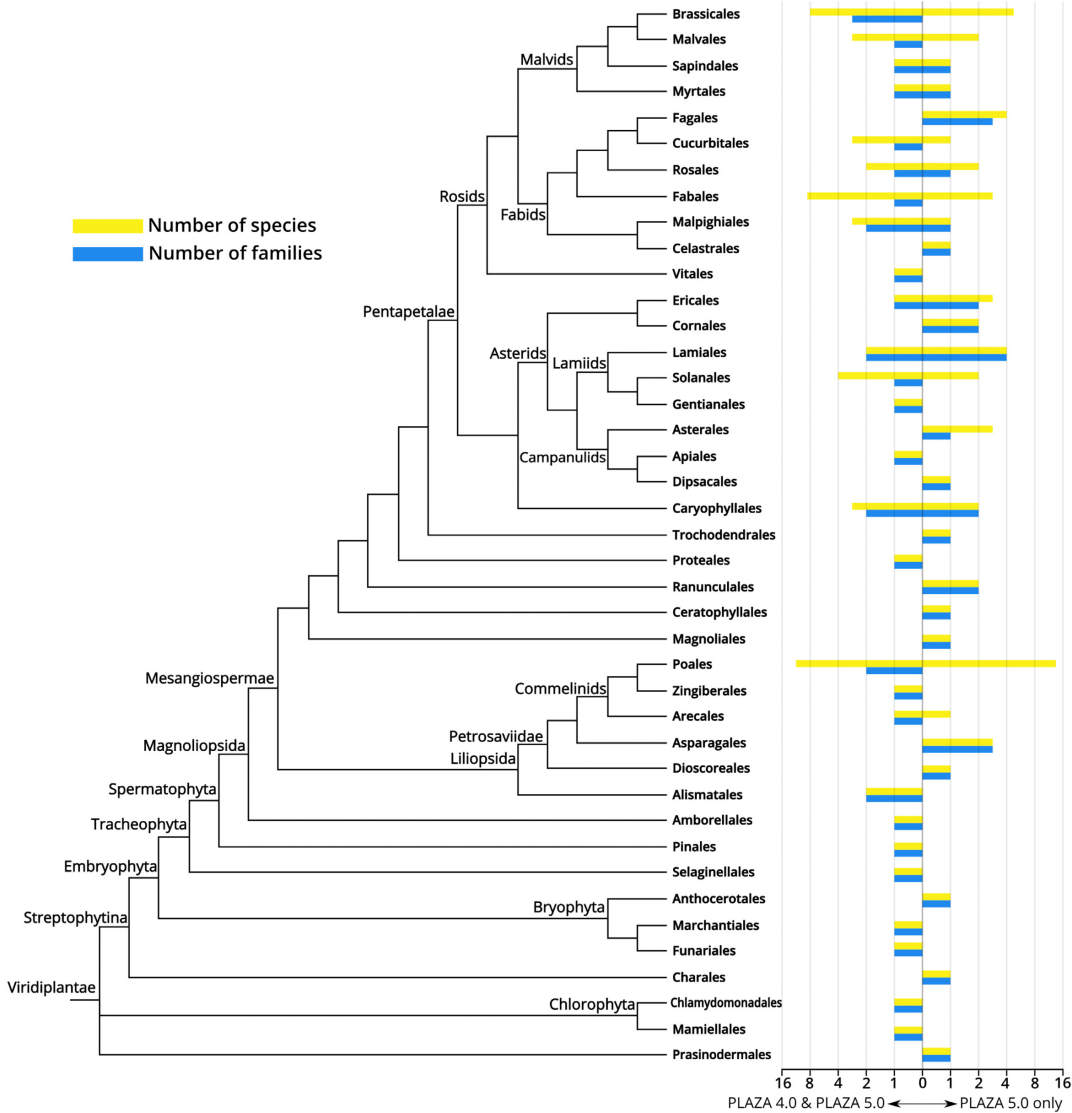
Species overview PLAZA 5.0. Overview of the species (yellow bars), taxonomic families (blue bars), and taxonomic orders (leaf nodes in the tree) in PLAZA 5.0 offset against the same content types in PLAZA 4.0. Bars that are solely to the left of the center indicate that there is no increase in PLAZA 5.0, while bars that are solely to the right of the center indicate that this order was not previously present in PLAZA 4.0. Bars with content to both sides of the center indicate that the order has been extended by the addition of new species and/or families in PLAZA 5.0.

The overview of the taxonomic orders and families now included in PLAZA 5.0 (41 orders and 66 families) shows that the resolution of plant diversity has increased markedly compared to PLAZA 4.0 (see Figure 1). Almost all orders are covered by one or two families and a handful of species, with the one major exception being the Poales (2 families and 28 species). Because the Poales order contains numerous cereal crops cultivated around the world for food, fodder or industrial purposes, it comes as no surprise to find the most economically important species in this order. As such, the Poales order is highly overrepresented when it comes to the number of species in the Monocots.

For both PLAZA instances, we also constructed the associated species tree, which is a key entity within the platform to group species based on their evolutionary relatedness, and during the gene family tree reconciliation procedure, to aid the identification of paralogous and orthologous genes. A full description of the PLAZA build procedure is provided in Supplementary Method S1. We constructed the species trees with information derived from NCBI taxonomy, coupled with additional information from a variety of publications (see Supplementary Method S2).

### Data content

The PLAZA framework supports four different gene types: protein-coding genes, RNA genes, transposable elements, and pseudogenes. Whereas protein-coding genes are always annotated within genome annotation projects, the other gene types are often not or only partially annotated. Differences in the applied genome annotation approaches can be seen by plotting all gene types per species (see Supplementary Figure S1). However, as all comparative genomics analyses in PLAZA are performed solely on protein-coding genes, the (partial) absence of the other gene types for some species does not affect PLAZA’s global functionality.

The functional genomics features of the PLAZA framework accelerate research by providing the backbone for a wide variety of analyses. Within each PLAZA instance, the functional annotation of genes can be split into ontology/classification-based annotations and free-text annotations. While free-text descriptions of genes can be extensive and very detailed, they are only available for a small number of well-curated model plants (e.g. *A. thaliana, Z. mays*). Ontology- and classification-based annotations allow for automated analysis procedures and can be automatically generated for a large fraction of genes. The associated annotations in PLAZA are based on three different ontologies/classifications: the gene ontology (GO) (21), InterPro (22), and MapMan (23). GO annotations do not merely assign genes with a certain ontology term, but an evidence code is also provided, indicating the source and confidence in the annotation. We used InterProScan (22) to identify conserved domains in protein-coding genes, on which basis a select subset of GO annotations can be deduced. In addition, we parsed GO annotations from https://geneontology.org (21) and the Gene Ontology Annotation (GOA) project (24) in order to integrate GO annotations with experimental evidence codes as well (16 328 genes from 13 plant species). One final important step in the functional annotation part of the PLAZA build procedure is the projection of GO annotations. Empirically validated GO annotations, identified through their associated evidences codes (restricted to EXP, IDA, IPI, IMP, IGI, IEP, TAS, IC, HDA, HEP, HTP, HMP and HGI) are also assigned to a selected set of orthologs (1,2).

Table 1 gives an overview of the ontology-based annotations in PLAZA 5.0. The fractions of genes annotated with the different ontologies is very similar between PLAZA Dicots 5.0 and Monocots 5.0: InterPro (77% and 76% resp.), MapMan (43% and 41% resp.) and GO (70% in both instances). Not all ontologies do provide the same level of information, therefore the integration of InterPro, MapMan and GO annotations are complementarity to the functional toolkit of PLAZA, as different plant biological processes are captured.

**Table 1.** Overview of the functional annotation data content in PLAZA 5.0. The MapMan annotations with MapMan term ‘35’ (‘Not assigned’), or its hierarchical descendants, were excluded. The GO annotations with root-level GO terms (‘biological_process’, ‘cellular_component’, ‘molecular_function’) were excluded. Primary GO sources are: the genome project data provider, the GOA project, and data from GeneOntology. Empirical GO evidence types consist of experimental GO evidence types (EXP, IDA, IPI, IMP, IGI, IEP, HTP, HDA, HMP, HGI, HEP), and traceable/curated author statements (IC, TAS)

		PLAZA Dicots 5.0	PLAZA Monocots 5.0
Genes	All gene types	4 234 318	2 254 715
	Protein coding genes	3 667 693	2 165 730
Genes with InterPro annotations		2 808 722 (76.6%)	1 636 297 (75.5%)
Genes with MapMan annotations		1 578 937 (43%)	888 280 (41%)
Genes with GO annotations	All data sources	2 563 423 (69.9%)	1 515 180 (70%)
	Primary sources only	2 114 497 (57.6%)	1 242 612 (57.4%)
	PLAZA GO projection	1 573 218 (42.9%)	918 149 (42.4%)
	Empirical evidences only	76 230 (2%)	65 860 (3%)

## NEW FEATURES

### Import of genes in PLAZA workbench via BED files

**Figure 2.**
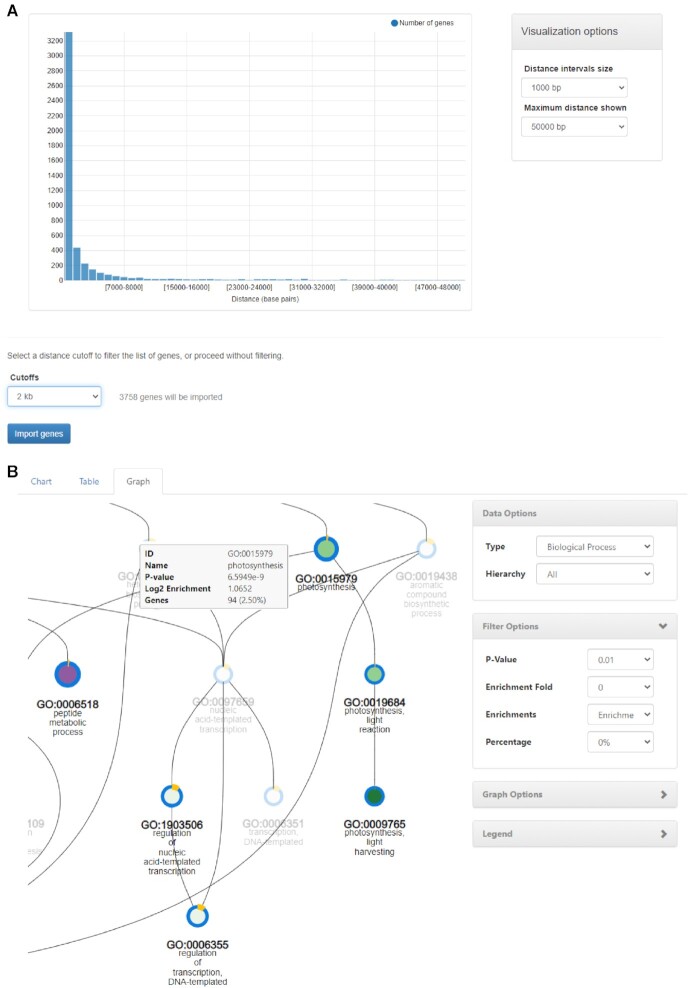
BED import and improved functional enrichment visualization. (**A**) After selecting the target species and gene types to be included (*Zea mays* B73, NAM v5.0), and uploading an example BED file (maize GLK2 ChIP-Seq (26)), each region is associated to the closest gene. Next, an interactive peak-to-gene distance distribution for all peaks and their associated closest genes is shown. A distance cutoff can be applied to retain all genes, or only regions overlapping with the gene body, or within a predefined distance to the closest gene. Applying a 2 kb cutoff retained 3758 genes in this workbench experiment. (**B**) Overview of a GO enrichment analysis for maize GLK2 ChIP-Seq target genes. Through the Graph visualization, the Filtered view discards parental GO terms and reports the most specific GO terms showing significant enrichment. While node size is proportional to the *P*-value of the enriched term, the node color is determined by the enrichment fold of the ontology term, with the color scale varying between green and purple (high and low values, depicting over-representation and depletion, respectively). The node outer band is determined by the percentage of genes that are annotated with the enriched ontology term (e.g. the selected node GO:0015979 reports that 2.50% of the genes in this workbench experiment are annotated with the enriched GO Biological Process term photosynthesis).

The PLAZA workbench is an important feature of the platform in which user-defined gene sets can be uploaded (called experiments). For each experiment, which co-workers can share between themselves via different types of access rights (e.g. Read or Edit permission), an extensive toolbox and various visualizations are available, as well as multiple export options. The workbench not only serves as a timesaving function where the end-user does not need to visit the pages of all genes of interest, but also offers different types of analyses that are not available when studying only a single gene. This includes functional analyses (e.g. functional enrichment through GO, MapMan or InterPro), structural analyses (e.g. viewing the genome-wide organization of a gene set), and data transformations (e.g. the translation of a gene set from one species to another species using PLAZA’s integrative orthology (25)). In previous PLAZA versions users could upload their gene sets through a list of (internal or external) gene identifiers or based on a sequence similarity search in which case the best-hit, from all or a selected species, was used to select a gene for each provided sequence. In PLAZA 5.0, we further extend the functionality of the workbench by also allowing users to load their genes by providing Browser Extensible Data (BED) files. BED files represent genomic locations for specific features, such as transcription factor (TF) – DNA interactions (ChIP-Seq), transcription start sites (CAGE), histone modifications or DNA methylation, or accessible chromatin (DNase/ATAC-Seq). By automatically mapping the regions in the BED file to the closest gene(s), with optional distance cutoffs applied by the end-user (see Figure 2A), PLAZA can now also function as part of workflows studying genomic regions derived from high-throughput sequencing experiments. As illustrated in Figure 2B, this new BED import function allows for the efficient functional exploration of, for example, TF binding data obtained through ChIP-Seq (26), generating insights in the biological processes controlled by specific regulators.

### Improved interactive visualization of functional enrichments

One of the key analyses performed when interpreting sets of genes that are expected to be co-regulated (e.g. differentially expressed genes), is whether certain functional annotations are statistically overrepresented within that gene set. The standard way to test this hypothesis is by applying an overrepresentation (or enrichment) analysis (27). In PLAZA 5.0, the workbench was extended to allow the enrichment procedure to be applied to not only the GO, but also InterPro and MapMan annotations. The procedure results in three different numeric values per enriched ontology term, which are key to correctly interpreting the result: the enrichment score representing the over-representation, the Bonferonni corrected *P*-value indicating the statistical significance, and the percent of genes annotated with that ontology term within the gene set. This last value, while often overlooked, is important in order not to over-interpret results when an enriched ontology term is associated with a very small number of genes in the dataset yet has a high enrichment score and low *P*-value.

The structure of GO can be described in terms of a graph, where each GO term is a node and the relationships between the terms are edges between the nodes. GO enrichment results can be redundant and complex to interpret due the wealth of parental GO terms, typically representing more general functional annotations. In order to allow the user to efficiently interpret GO enrichment results and focus on the most informative results, the resulting graph visualization of enriched GO terms has been significantly updated and made interactive (see Figure 2B). This redesigned graph display now conveys a broad variety of information, while still being able to be interpreted quickly: the node size is scaled by the *P*-value, the node color is determined by the enrichment score, and the node outer band represents the percentage of genes annotated with the enriched ontology term. Furthermore, different filtering options are available to select enriched GO terms from a specific aspect (molecular function, biological process, and cellular component) and focus on either over-represented or depleted GO terms. A similar graph display is also available to explore MapMan enrichment results.

### Genome-wide visualization of gene sets

While genomic clustering of genes belonging to the same biological pathway is commonly found in prokaryotes (28), such transcriptional operons are not present in most eukaryotes. Yet, clustering of functionally related genes has been described in eukaryotic genomes (29). Therefore, a common research question is whether there is a chromosomal locational bias for a set of functionally related genes. While PLAZA already identifies functional clusters, reporting statistically significant clusters of neighboring genes based on the similarity of functional annotations, it was not possible to browse and explore the organization of specific (functional) gene sets. In order to address this limitation, the latest release of the PLAZA platform now integrates Ideogram (https://eweitz.github.io/ideogram), which supports drawing and animating genome-wide datasets and offers the exploration of functional annotations, either GO, InterPro or MapMan, as well as gene sets from the Workbench (see Supplementary Figure S2).

### PLAZA REST API v3

Users could originally access the PLAZA platform either through the website, or through the data dumps on the FTP. In PLAZA 4.0, we introduced the PLAZA REST API, giving end-users more flexibility and options with regards to data retrieval. This PLAZA API (version 2) however had some shortcomings in functionality, and inconsistencies in the naming of the API function calls. We developed and designed the API v3, but will continue to support API v2 to accommodate existing workflows. We designed the new API to be consistent and easily extendable. More data types are accessible, and all returned content is structured in the same standardized way. Whereas in the API v2 only genes and gene families could be queried (in order to retrieve associated information), in API v3 this has been extended to functional ontologies: a standard example would be to retrieve all genes for a set of GO identifiers. Finally, the API also offers the capability to perform enrichment analyses, which thus allows end-users to work on sets of gene-sets, going one-step further than the capabilities of the workbench.

### Species instance finder

With the multitude of different PLAZA versions, and the associated multiple instances, it has become unwieldy for end-users to know which PLAZA instance is the one to use when they have a specific species in mind. This issue is further exacerbated by the multitude of assembly and annotation versions available for the majority of species. For example, for *A. thaliana* four different versions of the genome annotation are present in the different PLAZA versions and instances, while for *Oryza sativa ssp. japonica* five different genome annotations are available. In the PLAZA portal, we implemented a new tool, the Species Instance Finder, that allows the end-user to easily find all the instances that contain a given species, and allows additional filtering based on genome assembly and annotation version.

## CONCLUSION

The PLAZA platform continues to grow in both data content and features, keeping pace with the ever-increasing number of available plant genomes. This new release covers a wide taxonomic range of families, making the platform a valuable resource for comparative and evolutionary studies within the green plant lineage. The biodiversity of integrated genomes can benefit studies with an ecological or economic focus. The further development of the PLAZA workbench marks the continuous shift from single-gene to gene-set analyses.

## DATA AVAILABILITY

Users can access the PLAZA portal, with links to the PLAZA 5.0 instances described in this manuscript, at https://bioinformatics.psb.ugent.be/plaza/. Through the website all data can be accessed or downloaded, and bulk downloads are also available from our FTP server https://ftp.psb.ugent.be/pub/plaza/.

## Supplementary Material

gkab1024_Supplemental_File

## References

[1] Proost S. , Van Bel M., Sterck L., Billiau K., Van Parys T., Van de Peer Y., Vandepoele K. PLAZA: a comparative genomics resource to study gene and genome evolution in plants. Plant Cell. 2009; 21:3718–3731. 20040540 10.1105/tpc.109.071506 PMC2814516

[2] Van Bel M. , Proost S., Wischnitzki E., Movahedi S., Scheerlinck C., Van de Peer Y., Vandepoele K. Dissecting plant genomes with the PLAZA comparative genomics platform. Plant Physiol. 2012; 158:590–600. 22198273 10.1104/pp.111.189514 PMC3271752

[3] Shirasawa K. , Harada D., Hirakawa H., Isobe S., Kole C. Chromosome-level de novo genome assemblies of over 100 plant species. Breed Sci. 2021; 71:117–124. 34377059 10.1270/jsbbs.20146 PMC8329882

[4] Jung H. , Winefield C., Bombarely A., Prentis P., Waterhouse P. Tools and strategies for long-read sequencing and de novo assembly of plant genomes. Trends Plant Sci. 2019; 24:700–724. 31208890 10.1016/j.tplants.2019.05.003

[5] Valentin G. , Abdel T., Gaetan D., Jean-Francois D., Matthieu C., Mathieu R. GreenPhylDB v5: a comparative pangenomic database for plant genomes. Nucleic Acids Res. 2021; 49:D1464–D1471. 33237299 10.1093/nar/gkaa1068 PMC7779052

[6] Yates A.D. , Achuthan P., Akanni W., Allen J., Allen J., Alvarez-Jarreta J., Amode M.R., Armean I.M., Azov A.G., Bennett R. et al. Ensembl 2020. Nucleic Acids Res. 2020; 48:D682–D688. 31691826 10.1093/nar/gkz966 PMC7145704

[7] Goodstein D.M. , Shu S., Howson R., Neupane R., Hayes R.D., Fazo J., Mitros T., Dirks W., Hellsten U., Putnam N. et al. Phytozome: a comparative platform for green plant genomics. Nucleic Acids Res. 2012; 40:D1178–D1186. 22110026 10.1093/nar/gkr944 PMC3245001

[8] Cruz A. , Arrais J.P., Machado P. Interactive and coordinated visualization approaches for biological data analysis. Brief. Bioinform. 2019; 20:1513–1523. 29590305 10.1093/bib/bby019

[9] International Wheat Genome Sequencing, C. A chromosome-based draft sequence of the hexaploid bread wheat (Triticum aestivum) genome. Science. 2014; 345:1251788. 25035500 10.1126/science.1251788

[10] Wu S. , Wang X., Reddy U., Sun H., Bao K., Gao L., Mao L., Patel T., Ortiz C., Abburi V.L. et al. Genome of ‘Charleston Gray’, the principal American watermelon cultivar, and genetic characterization of 1,365 accessions in the U.S. National Plant Germplasm System watermelon collection. Plant Biotechnol. J. 2019; 17:2246–2258. 31022325 10.1111/pbi.13136 PMC6835170

[11] Valliyodan B. , Cannon S.B., Bayer P.E., Shu S., Brown A.V., Ren L., Jenkins J., Chung C.Y., Chan T.F., Daum C.G. et al. Construction and comparison of three reference-quality genome assemblies for soybean. Plant J. 2019; 100:1066–1082. 31433882 10.1111/tpj.14500

[12] Sork V.L. , Fitz-Gibbon S.T., Puiu D., Crepeau M., Gugger P.F., Sherman R., Stevens K., Langley C.H., Pellegrini M., Salzberg S.L. First draft assembly and annotation of the genome of a california endemic oak quercus lobata nee (Fagaceae). G3 (Bethesda). 2016; 6:3485–3495. 27621377 10.1534/g3.116.030411 PMC5100847

[13] Raymond O. , Gouzy J., Just J., Badouin H., Verdenaud M., Lemainque A., Vergne P., Moja S., Choisne N., Pont C. et al. The Rosa genome provides new insights into the domestication of modern roses. Nat. Genet. 2018; 50:772–777. 29713014 10.1038/s41588-018-0110-3 PMC5984618

[14] Cai L. , Arnold B.J., Xi Z., Khost D.E., Patel N., Hartmann C.B., Manickam S., Sasirat S., Nikolov L.A., Mathews S. et al. Deeply altered genome architecture in the endoparasitic flowering plant sapria himalayana griff (Rafflesiaceae). Curr. Biol. 2021; 31:1002–1011. 33485466 10.1016/j.cub.2020.12.045

[15] Yang R. , Jarvis D.E., Chen H., Beilstein M.A., Grimwood J., Jenkins J., Shu S., Prochnik S., Xin M., Ma C. et al. The reference genome of the halophytic plant eutrema salsugineum. Front. Plant Sci. 2013; 4:46. 23518688 10.3389/fpls.2013.00046 PMC3604812

[16] Yoshida S. , Kim S., Wafula E.K., Tanskanen J., Kim Y.M., Honaas L., Yang Z., Spallek T., Conn C.E., Ichihashi Y. et al. Genome sequence of striga asiatica provides insight into the evolution of plant parasitism. Curr. Biol. 2019; 29:3041–3052. 31522940 10.1016/j.cub.2019.07.086

[17] Rensing S.A. Why we need more non-seed plant models. New Phytol. 2017; 216:355–360. 28191633 10.1111/nph.14464

[18] Van Bel M. , Bucchini F., Vandepoele K. Gene space completeness in complex plant genomes. Curr. Opin. Plant Biol. 2019; 48:9–17. 30797187 10.1016/j.pbi.2019.01.001

[19] Van Bel M. , Diels T., Vancaester E., Kreft L., Botzki A., Van de Peer Y., Coppens F., Vandepoele K. PLAZA 4.0: an integrative resource for functional, evolutionary and comparative plant genomics. Nucleic Acids Res. 2018; 46:D1190–D1196. 29069403 10.1093/nar/gkx1002 PMC5753339

[20] Michael T.P. , VanBuren R. Building near-complete plant genomes. Curr. Opin. Plant Biol. 2020; 54:26–33. 31981929 10.1016/j.pbi.2019.12.009

[21] Gene Ontology, C. The Gene Ontology resource: enriching a GOld mine. Nucleic Acids Res. 2021; 49:D325–D334. 33290552 10.1093/nar/gkaa1113 PMC7779012

[22] Blum M. , Chang H.Y., Chuguransky S., Grego T., Kandasaamy S., Mitchell A., Nuka G., Paysan-Lafosse T., Qureshi M., Raj S. et al. The InterPro protein families and domains database: 20 years on. Nucleic Acids Res. 2021; 49:D344–D354. 33156333 10.1093/nar/gkaa977 PMC7778928

[23] Schwacke R. , Ponce-Soto G.Y., Krause K., Bolger A.M., Arsova B., Hallab A., Gruden K., Stitt M., Bolger M.E., Usadel B. MapMan4: a refined protein classification and annotation framework applicable to multi-omics data analysis. Mol Plant. 2019; 12:879–892. 30639314 10.1016/j.molp.2019.01.003

[24] Huntley R.P. , Sawford T., Mutowo-Meullenet P., Shypitsyna A., Bonilla C., Martin M.J., O’Donovan C. The GOA database: gene Ontology annotation updates for 2015. Nucleic Acids Res. 2015; 43:D1057–D1063. 25378336 10.1093/nar/gku1113 PMC4383930

[25] Vandepoele K. A Guide to the PLAZA 3.0 plant comparative genomic database. Methods Mol. Biol. 2017; 1533:183–200. 27987171 10.1007/978-1-4939-6658-5_10

[26] Tu X. , Mejia-Guerra M.K., Valdes Franco J.A., Tzeng D., Chu P.Y., Shen W., Wei Y., Dai X., Li P., Buckler E.S. et al. Reconstructing the maize leaf regulatory network using ChIP-seq data of 104 transcription factors. Nat. Commun. 2020; 11:5089. 33037196 10.1038/s41467-020-18832-8 PMC7547689

[27] Bauer S. Gene-category analysis. Methods Mol. Biol. 2017; 1446:175–188. 27812943 10.1007/978-1-4939-3743-1_13

[28] Osbourn A.E. , Field B. Operons. Cell. Mol. Life Sci. 2009; 66:3755–3775. 19662496 10.1007/s00018-009-0114-3 PMC2776167

[29] Lee J.M. , Sonnhammer E.L. Genomic gene clustering analysis of pathways in eukaryotes. Genome Res. 2003; 13:875–882. 12695325 10.1101/gr.737703 PMC430880

