# Supplementary material for: PLAZA 5.0: extending the scope and power of comparative and functional genomics in plants
Source: Nucleic Acids Res. 2021 Nov 8;50(D1):D1468–74. doi: 10.1093/nar/gkab1024 (PMC8728282; doi:10.1093/nar/gkab1024)
Supplement: gkab1024_Supplemental_File [file gkab1024_supplemental_file.pdf]

# Supplementary information

## PLAZA 5.0: extending the scope and power of comparative and functional genomics in plants

Michiel Van Bel[1,2], Francesca Silvestri[1,2], Lukasz Kreft[3], Alexander Botzki[4], Eric M. Weitz[5], Frederik Coppens[1,2], Klaas Vandepoele[1,2,6,*]

[1] Department of Plant Biotechnology and Bioinformatics, Ghent University, Technologiepark 71, 9052 Ghent, Belgium
[2] VIB Center for Plant Systems Biology, Technologiepark 71, 9052 Ghent, Belgium
[3] Institute of Biochemistry and Biophysics, Polish Academy of Sciences, Pawińskiego 5A 02-106 Warsaw, Poland
[4] VIB Bioinformatics Core, 9052 Ghent, Belgium
[5] Data Sciences Platform, Broad Institute of MIT and Harvard, Cambridge, MA 02142, USA
[6] Bioinformatics Institute Ghent, Ghent University, Technologiepark 71, 9052 Ghent, Belgium

* Corresponding author: klaas.vandepoele@psb.vib-ugent.be

# Supplementary Methods

## Supplementary Method S1: PLAZA 5.0 build procedure

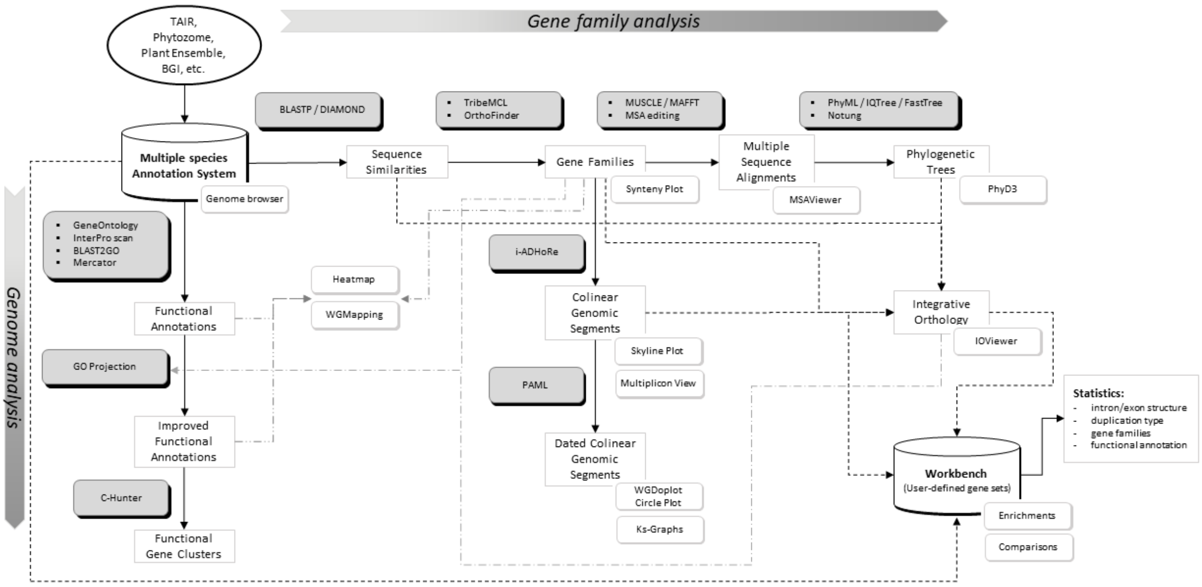


Flowchart depicting the different steps used to build the PLAZA platform. Grey boxes represent methods & tools whereas white shadowed boxes refer to website visualization tools. A detailed description of the construction steps and program settings is given below.

Data retrieval and delineation of gene families

All gene annotations were retrieved from the different data providers (for details, see section Data content in PLAZA Documentation) and stored according to their gene type (protein-coding, RNA, pseudo-gene and transposon). When parsing the structural gene annotation we verified if the original gene coordinates generate the correct transcript and protein sequence (as reported by the primary data) and flagged incorrect gene models.

Starting from all protein-coding genes, only retaining the longest transcript if alternative splicing variants exist, protein sequences were used to construct gene families by applying sequence-based protein clustering. First, an all-against-all sequence comparison was performed using v0.9.30 of DIAMOND (1), applying an E-value threshold of 1e-05 and retaining the best 2500 hits. Next, the clustering of protein coding genes into homologous (HOM) gene families was performed using v14.137 of Tribe-MCL (2) (mclblastline, default parameters except I = 2 and scheme = 4), while the clustering into orthologous (ORTHO) families was performed using v1.1.10 of OrthoFinder (3).

Detection of colinearity

Colinearity within and between species was identified using v3.0.01 of i-ADHoRe (4), which detects genomic homology based on the identification of conservation of gene content and gene order. i-ADHoRe was run with the following settings: alignment_method gg2, gap_size 30, cluster_gap 35, q_value 0.85, prob_cutoff 0.01, anchor_points 5 and level_2_only true. A default run, including all organisms, is provided.

Multiple sequence alignments and phylogenetic trees

For all gene families, multiple sequence alignments were created using v7.453 MAFFT (5). Alignment columns containing gaps were removed when a gap was present in >10% of the sequences. To reduce the chance of including misaligned amino acids, all positions in the alignment left or right from the gap were also removed until a column in the sequence alignment was found where the residues were conserved in all genes included in our analyses. This was determined as follows: for every pair of residues in the column, the BLOSUM62 value was retrieved. Next, the median value for all these values was calculated. If this median was ≥0, the column was considered as containing homologous amino acids. To prevent the emergence of long-branch attraction or badly-supported nodes yielding uninformative trees, highly divergent and partial sequences were removed from the alignment prior to phylogenetic tree construction, if they contained gaps in more than 50% of the alignment columns, or two times or more the number of gaps than the average number of gaps per sequence in the alignment.

Phylogenetic trees were constructed using v2.1.10 of FastTree (6) applying the JTT substitution model, 100 bootstrap samples, estimated proportion of invariable sites, four substitution categories, estimated gamma distribution parameter, the BIONJ distance-based tree as starting tree and without tree optimization (default parameters for protein sequences). NOTUNG 2.6 (7) was used to root the trees and to infer speciation and duplication events using the tree reconciliation mode and applying the Duplication/Loss Score to evaluate alternate hypotheses. PhyD3 (8) was used for interactive tree visualization.

Integrative Orthology

To infer orthologous gene relationships with an enhanced robustness, an integrative approach was developed to identify orthologs on a gene-by-gene basis (9). The developed ensemble approach consists of a combination of four distinct orthology prediction methods: orthologous gene families, reconciled phylogenetic trees, protein alignment scores and colinearity information. The integration of gene colinearity facilitates the detection of positional orthologs, namely genes with conserved genome organization between species. For each orthology relationship then, three different gene sequence features were also compared: protein-coding sequence (CDS) length, number of exons and GC content.

Functional Annotations

The InterPro and GeneOntology ontologies were retrieved from the respective host websites: version 86.0 for InterPro and release 2021-07-01 for GeneOntology. InterPro scan v5.24-63 (10) was run on all protein-coding genes, and additional GO annotations were inferred with InterPro-to-GO mapping. Additional GO annotations were retrieved from the genome projects where available, as well as from http://geneontology.org (11,12) and from the GO Annotation (GOA) project (13). MapMan annotations were provided by Björn Usadel and Marie Bolger, using Mercator 4 (14) to generate the annotations.

Redundant GO annotations were merged according to the GO evidence code rank (15). To avoid the inclusion of obsolete GO terms, a filter was applied using the set of valid GO terms derived from the GO OBO file (v1.2).

The GO terms were also projected, assigning empirically validated GO annotations to a selected set of orthologs (9,16,17). GO annotations from (mainly) genes from well-studied model organisms are transferred to their orthologous counterparts in other species, where repeating the experiments necessary to gain (the same) knowledge would take too much time and effort. In order to minimize the number of false positives after the GO projection,

limitations are placed on two parts of the transfer operation. First, only experimentally validated GO annotations can be projected, thus removing the possible effects of cascading error. Secondly, the methodology used for orthology delineation is quite restrictive.

GO enrichment was performed for each gene family, with only the organisms with genes in the gene family under investigation being used as the background model for the statistical analysis (hypergeometric distribution with Bonferroni correction for multiple testing). Only GO terms covering at least half of the annotated genes in a family and with corrected values of P <= 0.05 were retained.

Functional Gene Clusters

To study the clustering of functionally related genes (functional clusters), the C-Hunter program (18) was used for a genome-wide analysis. This tool detects statistically significant clusters of neighboring genes based on the similarity of GO annotations. The settings that were used are: minimum and maximum cluster sizes of 2/30 and 10/150 respectively, e-value cutoff of 0.001 and 50% maximum cluster overlap.

## Supplementary Method S2: Species tree construction

The species trees for both the PLAZA Monocots 5.0 and PLAZA Dicots 5.0 instances were constructed in the following way:

1. We provided the NCBI taxonomy identifiers of all species in the PLAZA instances to the ENA API (https://www.ebi.ac.uk/ena/browser/api/) in order to retrieve the associated lineage information (i.e. the parental nodes and associated NCBI taxonomy identifiers).
2. We used this information to automatically generate the first rudimentary species trees, which, unfortunately, has quite some limitations: a large number of speciation events cannot be resolved, leading to many multifurcations.
3. We then manually resolved the multifurcations in these intermediate species trees using a wide variety of publications:
    - Grouping of mosses, liverworts, and hornworts (19,20)
    - Resolving Magnoliidae and Ceratophyllales in relationship to eudicots and monocots (20-22)
    - Resolving placement of Dioscorea dumetorum (20)
    - Resolving trifurcation in Asparagales (23)
    - Resolving Musa, Poales, and Arecales trifurcation (24)
    - Resolving trifurcation in Chloridoideae (25)
    - Resolving trifurcation in Andropogoneae (26)
    - Resolving trifurcation in Paniceae (27)
    - Resolving trifurcation in Pooideae (28)
    - Resolving trifurcation in BOPClade (29)
    - Resolving multifurcations in Brassicaceae (30)
    - Resolving multifurcations in Malvaceae (31)
    - Resolving multifurcations in Malvids (32)
    - Resolving multifurcations in IRL-clade (33)
    - Resolving multifurcations in Phaseoleae (33)
    - Resolving multifurcations in Malpighiales (34)
    - Resolving multifurcations in 50_kb_inversion_clade (35)
    - Resolving multifurcations in fabids (36)
    - Resolving multifurcations in Solanaceae (37)
    - Resolving multifurcations in Lamiales (38)
    - Resolving multifurcations in Lamiids (39)
    - Resolving multifurcations in Campanulids (40)
    - Resolving multifurcations in Caryophyllales (41)
    - Resolving multifurcations in Asterids (42)
    - Resolving multifurcations in Mesangiospermae (43)

# Supplementary Tables

## Supplementary Table S1: Data content and sources PLAZA 5.0

| PLAZA identifier | Common Name | Taxonomy ID | Pubmed ID | Reference | Dicots 5.0 | Monocots 5.0 |
|---|---|---|---|---|---|---|
| acertr | *Acer truncatum* | 47965 | 32772482 | (44) | ✓ | |
| ach | *Actinidia chinensis* | 3625 | 31645971 | (45) | ✓ | |
| ata | *Aegilops tauschii* | 37682 | 29143815 | (46) | | ✓ |
| aar | *Aethionema arabicum* | 228871 | 31554715 | (47) | ✓ | |
| asa | *Allium sativum* | 4682 | 32730994 | (23) | | ✓ |
| amhyb | *Amaranthus hybridus* | 3565 | 32835372 | (48) | ✓ | |
| atr | *Amborella trichopoda* | 13333 | 24357323 | (49) | ✓ | ✓ |
| aco | *Ananas comosus* | 4615 | 26523774 | (50) | | ✓ |
| aag | *Anthoceros agrestis* | 41834 | 32170292 | (19) | ✓ | ✓ |
| aox | *Aquilegia oxysepala [kansuensis]* | 432649 | 32637141 | (51) | ✓ | |
| aly | *Arabidopsis lyrata* | 59689 | 26382944 | (52) | ✓ | |
| ath | *Arabidopsis thaliana [COL-0]* | 3702 | 27862469 | (53) | ✓ | ✓ |
| arhy | *Arachis hypogaea [Tifrunner]* | 3818 | 31043755 | (54) | ✓ | |
| aof | *Asparagus officinalis* | 4686 | 29093472 | (55) | | ✓ |
| ama | *Avicennia marina* | 82927 | 33561229 | (56) | ✓ | |
| bvu | *Beta vulgaris* | 161934 | *NA* | (57) | ✓ | |
| bdi | *Brachypodium distachyon* | 15368 | 20148030 | (58) | | ✓ |
| bca | *Brassica carinata* | 52824 | 33599732 | (59) | ✓ | |
| bna | *Brassica napus [Darmor-bzh]* | 3708 | 33319912 | (60) | ✓ | |
| bol | *Brassica oleracea [HDEM]* | 109376 | 30390080 | (61) | ✓ | |
| bra | *Brassica rapa [Z1]* | 3711 | 30390080 | (61) | ✓ | |
| calsi | *Calamus simplicifolius* | 746888 | 30101322 | (62) | | ✓ |
| camsi | *Camellia sinensis var. sinensis [Shuchazao]* | 542762 | 32353625 | (63) | ✓ | |
| cansat | *Cannabis sativa* | 3483 | 32377363 | (64) | ✓ | |
| cru | *Capsella rubella* | 81985 | 23749190 | (65) | ✓ | |
| can | *Capsicum annuum* | 4072 | 24441736 | (66) | ✓ | |
| chi | *Cardamine hirsuta* | 50463 | 27797353 | (67) | ✓ | |
| cpa | *Carica papaya* | 3649 | 18432245 | (68) | ✓ | |
| cfa | *Carpinus fangiana* | 176857 | 31964866 | (69) | ✓ | |
| cil | *Carya illinoinensis [Pawnee]* | 32201 | 34226565 | (70) | ✓ | |
| cpu | *Cenchrus purpureus* | 154765 | 33040437 | (71) | | ✓ |
| cde | *Ceratophyllum demersum* | 4428 | 32094642 | (22) | ✓ | ✓ |
| cbr | *Chara braunii* | 69332 | 30007417 | (72) | ✓ | ✓ |
| cqu | *Chenopodium quinoa* | 63459 | 28178233 | (73) | ✓ | |
| cre | *Chlamydomonas reinhardtii* | 3055 | 17932292 | (74) | ✓ | ✓ |
| car | *Cicer arietinum L. [ICC 4958]* | 3827 | 26259924 | (75) | ✓ | |
| cla | *Citrullus lanatus [Charleston Gray]* | 3654 | 31022325 | (76) | ✓ | |
| ccl | *Citrus clementina* | 85681 | 24908277 | (77) | ✓ | |
| ccan | *Coffea canephora* | 49390 | 25190796 | (78) | ✓ | |
| col | *Corchorus olitorius* | 93759 | 28134914 | (79) | ✓ | |
| cav | *Corylus avellana [Tonda Gentile delle Langhe]* | 13451 | 33964151 | (80) | ✓ | |

| | | | | | | |
|---|---|---|---|---|---|---|
| cme | *Cucumis melo* | 3656 | 22753475 | (81) | ✓ | |
| csa | *Cucumis sativus L. [Chinese Long]* | 3659 | 31216035 | (82) | ✓ | |
| dca | *Daucus carota* | 4039 | 27158781 | (83) | ✓ | |
| din | *Davidia involucrata* | 16924 | 31970919 | (84) | ✓ | |
| dex | *Digitaria exilis* | 1010633 | 32901040 | (85) | | ✓ |
| ddu | *Dioscorea dumetorum* | 167584 | 32143301 | (86) | | ✓ |
| dzi | *Durio zibethinus* | 66656 | 28991254 | (31) | ✓ | |
| egu | *Elaeis guineensis* | 51953 | 23883927 | (87) | | ✓ |
| ecu | *Eragrostis curvula* | 38414 | 31308395 | (88) | | ✓ |
| eca | *Erigeron canadensis* | 72917 | 31951071 | (89) | ✓ | |
| egut | *Erythranthe guttata* | 4155 | 24225854 | (90) | ✓ | |
| egr | *Eucalyptus grandis* | 71139 | 24919147 | (91) | ✓ | |
| esa | *Eutrema salsugineum* | 72664 | 23518688 | (92) | ✓ | |
| fve | *Fragaria vesca* | 57918 | 31069085 | (93) | ✓ | |
| fan | *Fragaria x ananassa* | 3747 | 30804557 | (94) | ✓ | |
| gma | *Glycine max* | 3847 | 31433882 | (95) | ✓ | ✓ |
| ghi | *Gossypium hirsutum* | 3635 | 32313247 | (96) | ✓ | |
| gra | *Gossypium raimondii* | 29730 | 22922876 | (97) | ✓ | |
| han | *Helianthus annuus* | 4232 | 28538728 | (98) | ✓ | |
| hvu | *Hordeum vulgare* | 4513 | 33239781 | (99) | | ✓ |
| hma | *Hydrangea macrophylla* | 23110 | 33175097 | (100) | ✓ | |
| lsa | *Lactuca sativa* | 4236 | 28401891 | (101) | ✓ | |
| lpe | *Lolium perenne* | 4522 | 26408275 | (102) | | ✓ |
| lonja | *Lonicera japonica* | 105884 | 32187685 | (103) | ✓ | |
| lja | *Lotus japonicus [MG-20]* | 34305 | 32365501 | (104) | ✓ | |
| lal | *Lupinus albus* | 3870 | 31980615 | (105) | ✓ | |
| mbi | *Magnolia biondii* | 86725 | 33642574 | (21) | ✓ | ✓ |
| mdo | *Malus domestica* | 3750 | 28581499 | (106) | ✓ | |
| mes | *Manihot esculenta [Crantz]* | 3983 | 25504737 | (107) | ✓ | |
| mpo | *Marchantia polymorpha* | 3197 | 28985561 | (108) | ✓ | ✓ |
| mtr | *Medicago truncatula* | 3880 | 22089132 | (109) | ✓ | |
| mco | *Micromonas commoda* | 296587 | 19359590 | (110) | ✓ | ✓ |
| msi | *Miscanthus sinensis* | 62337 | 33116128 | (111) | | ✓ |
| mac | *Musa acuminata [banksii]* | 4641 | 22801500 | (112) | | ✓ |
| nnu | *Nelumbo nucifera [China antique]* | 4432 | 33514746 | (113) | ✓ | |
| nta | *Nicotiana tabacum* | 4097 | 28625162 | (114) | ✓ | |
| oeu | *Olea europaea* | 4146 | 27346392 | (115) | ✓ | |
| oth | *Oropetium thomaeum* | 1148796 | 31245697 | (116) | | ✓ |
| oal | *Oryza alta* | 52545 | 33539781 | (117) | | ✓ |
| obr | *Oryza brachyantha* | 4533 | 23518283 | (118) | | ✓ |
| osak | *Oryza sativa Kitaake [KitaakeX]* | 4530 | 31775618 | (119) | | ✓ |
| osaindica | *Oryza sativa ssp. indica* | 39946 | 11935017 | (120) | | ✓ |
| osa | *Oryza sativa ssp. Japonica* | 39947 | 16100779 | (121) | ✓ | ✓ |
| pha | *Panicum hallii* | 206008 | 30523281 | (122) | | ✓ |
| pso | *Papaver somniferum* | 3469 | 30166436 | (123) | ✓ | |
| pax | *Petunia axillaris* | 33119 | 27255838 | (124) | ✓ | |
| pvu | *Phaseolus vulgaris* | 3885 | *NA* | (125) | ✓ | |
| ped | *Phyllostachys edulis* | 38705 | 30202850 | (126) | | ✓ |

| | | | | | | |
|---|---|---|---|---|---|---|
| ppa | *Physcomitrium patens* | 3218 | 18079367 | (127) | ✓ | ✓ |
| psa | *Pisum sativum* | 3888 | 31477930 | (33) | ✓ | |
| ptr | *Populus trichocarpa* | 3694 | 16973872 | (128) | | ✓ |
| ptr | *Populus trichocarpa* | 3694 | 16973872 | (128) | ✓ | |
| pco | *Prasinoderma coloniale* | 156133 | 32572216 | (129) | ✓ | ✓ |
| ppe | *Prunus persica* | 3760 | 23525075 | (130) | ✓ | |
| pgr | *Punica granatum* | 22663 | 28654223 | (131) | ✓ | |
| qlo | *Quercus lobata* | 97700 | 27621377 | (132) | ✓ | |
| rsi | *Rhododendron simsii* | 118357 | 33077749 | (133) | ✓ | |
| rch | *Rosa chinensis* | 74649 | 29713014 | (134) | ✓ | |
| ssp | *Saccharum spontaneum* | 62335 | 30297971 | (135) | | ✓ |
| sbr | *Salix brachista* | 2182728 | 31745089 | (136) | ✓ | |
| sbo | *Salvia bowleyana [Dunn]* | 424416 | 33634943 | (137) | ✓ | |
| shi | *Sapria himalayana* | 289637 | 33485466 | (34) | ✓ | |
| spa | *Schrenkiella parvula* | 98039 | 21822265 | (138) | ✓ | |
| sce | *Secale cereale* | 4550 | 33737755 | (139) | | ✓ |
| sed | *Sechium edule* | 184140 | 33517348 | (140) | ✓ | |
| smo | *Selaginella moellendorffii* | 88036 | 21551031 | (141) | ✓ | ✓ |
| sun | *Selenicereus undatus* | 176265 | 33750805 | (142) | ✓ | |
| sgi | *Sequoiadendron giganteum* | 99814 | 32948606 | (143) | ✓ | ✓ |
| sit | *Setaria italica* | 4555 | 22580950 | (144) | | ✓ |
| svi | *Setaria viridis* | 4556 | 33020633 | (145) | | ✓ |
| sci | *Simmondsia chinensis* | 3999 | 32195345 | (146) | ✓ | |
| sly | *Solanum lycopersicum* | 4081 | 22660326 | (147) | ✓ | ✓ |
| spe | *Solanum pennellii* | 28526 | 25064008 | (148) | ✓ | |
| stu | *Solanum tuberosum* | 4113 | 21743474 | (149) | ✓ | |
| sbi | *Sorghum bicolor* | 4558 | 19189423 | (150) | | ✓ |
| spo | *Spirodela polyrhiza* | 29656 | 24548928 | (151) | | ✓ |
| sas | *Striga asiatica* | 4170 | 31522940 | (152) | ✓ | |
| tha | *Tarenaya hassleriana* | 28532 | 23983221 | (153) | ✓ | |
| tca | *Theobroma cacao [Matina 1-6]* | 3641 | 23731509 | (154) | ✓ | |
| tpr | *Trifolium pratense* | 57577 | 26617401 | (155) | ✓ | |
| twi | *Tripterygium wilfordii* | 458696 | 32080175 | (156) | ✓ | |
| tae | *Triticum aestivum* | 4565 | 25035500 | (157) | | ✓ |
| tdi | *Triticum dicoccoides* | 85692 | 28684525 | (158) | | ✓ |
| ttu | *Triticum turgidum [Svevo]* | 4567 | 30962619 | (159) | | ✓ |
| tar | *Trochodendron aralioides* | 4407 | 31738437 | (160) | ✓ | |
| ugi | *Utricularia gibba* | 13748 | 28507139 | (161) | ✓ | |
| vma | *Vaccinium macrocarpon [Stevens]* | 13750 | 33643360 | (162) | ✓ | |
| vpl | *Vanilla planifolia [Daphna]* | 51239 | *NA* | (163) | ✓ | ✓ |
| vmu | *Vigna mungo* | 3915 | 32794377 | (164) | ✓ | |
| vvi | *Vitis vinifera* | 29760 | 17721507 | (165) | ✓ | |
| zma-b104 | *Zea mays [B104]* | 4577 | *NA* | (166) | | ✓ |
| zma | *Zea mays [B73]* | 4577 | 19965430 | (167) | ✓ | ✓ |
| zosmarina | *Zostera marina* | 29655 | 26814964 | (168) | | ✓ |
| zjn | *Zoysia japonica ssp. nagirizaki* | 309978 | 26975196 | (169) | | ✓ |

# Supplementary Figures

## Supplementary Figure S1: Species overview of PLAZA 5.0

Data content overview Monocot (A) and Dicots (B) instances PLAZA 5.0. Orphan gene families are single-copy species-specific genes. GO evidence groups are based on the following GO evidence types: Experimental (EXP, IDA, IPI, IMP, IGI, IEP), Computational Reviewed (ISS, ISO, ISA, ISM, IGC, IBA, IBD, IKR, IRD, RCA) and Electronic (TAS, NAS, IC, ND, IEA, NR).

**A**

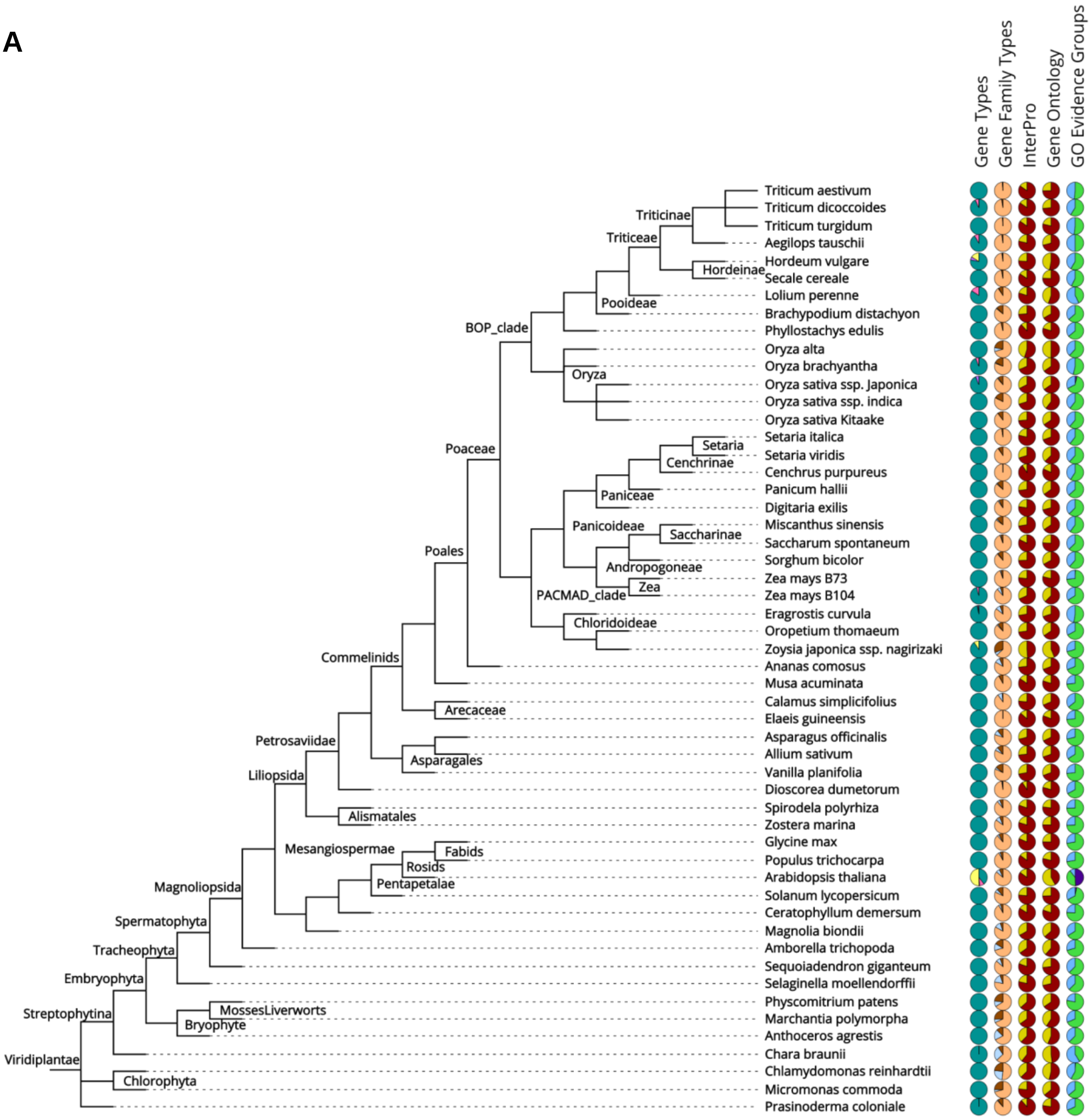

B

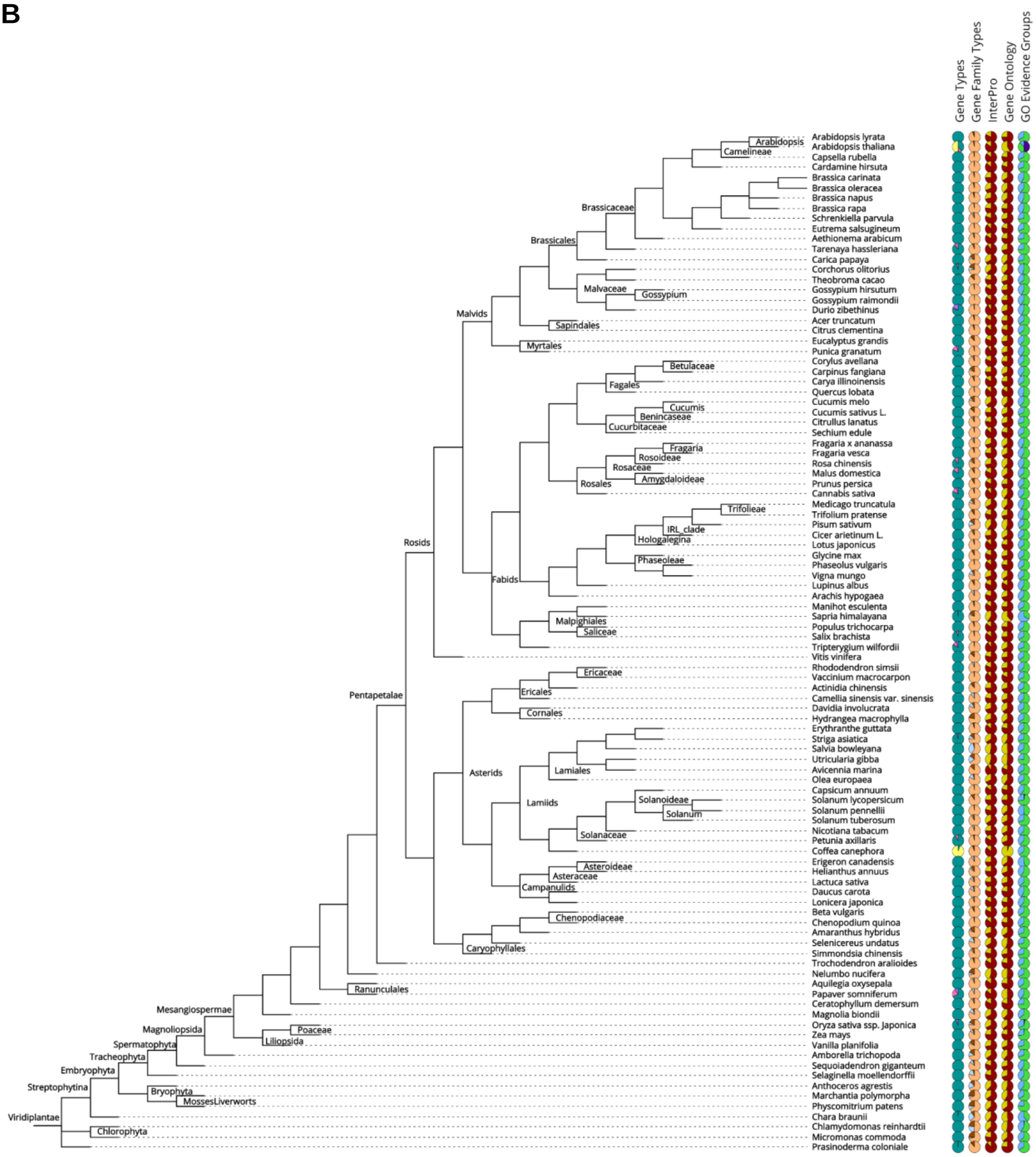
Gene Types
Gene Family Types
InterPro
Gene Ontology
GO Evidence Groups
Arabidopsis
Camelineae
Brassicaceae
Brassicales
Malvaceae
Gossypium
Malvids
Sapindales
Myrtales
Betulaceae
Fagales
Cucumis
Benincaseae
Cucurbitaceae
Fragaria
Rosoideae
Rosaceae
Amygdaloideae
Rosales
Trifolieae
IRL_clade
Hologalegina
Phaseoleae
Rosids
Fabids
Malpighiales
Saliceae
Ericaceae
Ericales
Pentapetalae
Cornales
Asterids
Lamiales
Lamiids
Solanoideae
Solanum
Solanaceae
Asteroideae
Asteraceae
Campanulids
Chenopodiaceae
Caryophyllales
Ranunculales
Mesangiospermae
Magnoliopsida
Poaceae
Spermatophyta
Liliopsida
Tracheophyta
Embryophyta
Streptophytina
Bryophyta
MossesLiverworts
Viridiplantae
Chlorophyta
Arabidopsis lyrata
Arabidopsis thaliana
Capsella rubella
Cardamine hirsuta
Brassica carinata
Brassica oleracea
Brassica napus
Brassica rapa
Schrenkiella parvula
Eutrema salsugineum
Aethionema arabicum
Tarenaya hassleriana
Carica papaya
Corchorus olitorius
Theobroma cacao
Gossypium hirsutum
Gossypium raimondii
Durio zibethinus
Acer truncatum
Citrus clementina
Eucalyptus grandis
Punica granatum
Corylus avellana
Carpinus fangiana
Carya illinoinensis
Quercus lobata
Cucumis melo
Cucumis sativus L.
Citrullus lanatus
Sechium edule
Fragaria x ananassa
Fragaria vesca
Rosa chinensis
Malus domestica
Prunus persica
Cannabis sativa
Medicago truncatula
Trifolium pratense
Pisum sativum
Cicer arietinum L.
Lotus japonicus
Glycine max
Phaseolus vulgaris
Vigna mungo
Lupinus albus
Arachis hypogaea
Manihot esculenta
Sapria himalayana
Populus trichocarpa
Salix brachista
Tripterygium wilfordii
Vitis vinifera
Rhododendron simsii
Vaccinium macrocarpon
Actinidia chinensis
Camellia sinensis var. sinensis
Davidia involucrata
Hydrangea macrophylla
Erythranthe guttata
Striga asiatica
Salvia bowleyana
Utricularia gibba
Avicennia marina
Olea europaea
Capsicum annuum
Solanum lycopersicum
Solanum pennellii
Solanum tuberosum
Nicotiana tabacum
Petunia axillaris
Coffea canephora
Erigeron canadensis
Helianthus annuus
Lactuca sativa
Daucus carota
Lonicera japonica
Beta vulgaris
Chenopodium quinoa
Amaranthus hybridus
Selenicereus undatus
Simmondsia chinensis
Trochodendron aralioides
Nelumbo nucifera
Aquilegia oxysepala
Papaver somniferum
Ceratophyllum demersum
Magnolia biondii
Oryza sativa ssp. Japonica
Zea mays
Vanilla planifolia
Amborella trichopoda
Sequoiadendron giganteum
Selaginella moellendorffii
Anthoceros agrestis
Marchantia polymorpha
Physcomitrium patens
Chara braunii
Chlamydomonas reinhardtii
Micromonas commoda
Prasinoderma coloniale

**Supplementary Figure S2: WGMapping tool**

Ideogram genome-mapping of two different gene sets in *Zea mays*. The red genes are a set of 3322 genes from the maize GLK2 ChIP-Seq experiment, obtained from the BED import example described in the main manuscript (Figure 2). The blue genes represent 445 genes annotated with the photosynthesis GO term (GO:0015979).

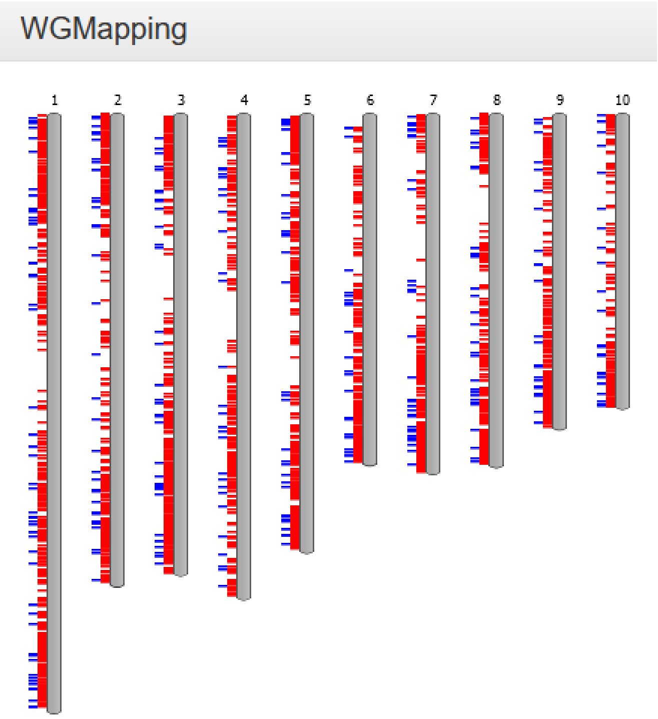

# References


1. Buchfink B, Xie C, Huson DH. Fast and sensitive protein alignment using DIAMOND. Nature methods. 2015 Jan;12(1):59–60.
2. Enright AJ, Van Dongen S, Ouzounis CA. An efficient algorithm for large-scale detection of protein families. Nucleic acids research. 2002 Apr 1;30(7):1575–84.
3. Emms DM, Kelly S. OrthoFinder: solving fundamental biases in whole genome comparisons dramatically improves orthogroup inference accuracy. Genome biology. 2015 Aug 6;16:157.
4. Proost S, Fostier J, De Witte D, Dhoedt B, Demeester P, Van de Peer Y, et al. i-ADHoRe 3.0—fast and sensitive detection of genomic homology in extremely large data sets. Nucleic Acids Research. 2012 Jan 1;40(2):e11–e11.
5. Nakamura T, Yamada KD, Tomii K, Katoh K. Parallelization of MAFFT for large-scale multiple sequence alignments. Bioinformatics. 2018 Jul 15;34(14):2490–2.
6. Price MN, Dehal PS, Arkin AP. FastTree 2--approximately maximum-likelihood trees for large alignments. PloS one. 2010 Mar 10;5(3):e9490.
7. Chen K, Durand D, Farach-Colton M. NOTUNG: a program for dating gene duplications and optimizing gene family trees. Journal of computational biology : a journal of computational molecular cell biology. 2000;7(3–4):429–47.
8. Kreft L, Botzki A, Coppens F, Vandepoele K, Van Bel M. PhyD3: a phylogenetic tree viewer with extended phyloXML support for functional genomics data visualization. Bioinformatics (Oxford, England). 2017 Sep 15;33(18):2946–7.
9. Van Bel M, Proost S, Wischnitzki E, Movahedi S, Scheerlinck C, Van de Peer Y, et al. Dissecting Plant Genomes with the PLAZA Comparative Genomics Platform1[W]. Plant Physiol. 2012 Feb;158(2):590–600.
10. Jones P, Binns D, Chang H-Y, Fraser M, Li W, McAnulla C, et al. InterProScan 5: genome-scale protein function classification. Bioinformatics. 2014 May 1;30(9):1236–40.
11. Ashburner M, Ball CA, Blake JA, Botstein D, Butler H, Cherry JM, et al. Gene Ontology: tool for the unification of biology. Nat Genet. 2000 May;25(1):25–9.
12. Gene Ontology Consortium. The Gene Ontology resource: enriching a GOld mine. Nucleic Acids Res. 2021 Jan 8;49(D1):D325–34.
13. Huntley RP, Sawford T, Mutowo-Meullenet P, Shypitsyna A, Bonilla C, Martin MJ, et al. The GOA database: gene Ontology annotation updates for 2015. Nucleic acids research. 2015 Jan;43(Database issue):D1057-63.
14. Schwacke R, Ponce-Soto GY, Krause K, Bolger AM, Arsova B, Hallab A, et al. MapMan4: A Refined Protein Classification and Annotation Framework Applicable to Multi-Omics Data Analysis. Molecular plant. 2019 Jun 3;12(6):879–92.
15. Buza TJ, McCarthy FM, Wang N, Bridges SM, Burgess SC. Gene Ontology annotation quality analysis in model eukaryotes. Nucleic Acids Research. 2008 Feb 1;36(2):e12.
16. Proost S, Van Bel M, Sterck L, Billiau K, Van Parys T, Van de Peer Y, et al. PLAZA: a comparative genomics resource to study gene and genome evolution in plants. The Plant cell. 2009 Dec;21(12):3718–31.
17. Proost S, Van Bel M, Vaneechoutte D, Van de Peer Y, Inzé D, Mueller-Roeber B, et al. PLAZA 3.0: an access point for plant comparative genomics. Nucleic Acids Research. 2015 Jan 28;43(D1):D974–81.
18. Yi G, Sze S-H, Thon MR. Identifying clusters of functionally related genes in genomes. Bioinformatics. 2007 May 1;23(9):1053–60.
19. Li F-W, Nishiyama T, Waller M, Frangedakis E, Keller J, Li Z, et al. Anthoceros genomes illuminate the origin of land plants and the unique biology of hornworts. Nat Plants. 2020 Mar;6(3):259–72.
20. One Thousand Plant Transcriptomes Initiative. One thousand plant transcriptomes and the phylogenomics of green plants. Nature. 2019 Oct;574(7780):679–85.

21. Dong S, Liu M, Liu Y, Chen F, Yang T, Chen L, et al. The genome of Magnolia biondii Pamp. provides insights into the evolution of Magnoliales and biosynthesis of terpenoids. Hortic Res. 2021 Mar 1;8(1):38.
22. Yang Y, Sun P, Lv L, Wang D, Ru D, Li Y, et al. Prickly waterlily and rigid hornwort genomes shed light on early angiosperm evolution. Nat Plants. 2020 Mar;6(3):215–22.
23. Sun X, Zhu S, Li N, Cheng Y, Zhao J, Qiao X, et al. A Chromosome-Level Genome Assembly of Garlic (Allium sativum) Provides Insights into Genome Evolution and Allicin Biosynthesis. Mol Plant. 2020 Sep 7;13(9):1328–39.
24. Barrett CF, Baker WJ, Comer JR, Conran JG, Lahmeyer SC, Leebens-Mack JH, et al. Plastid genomes reveal support for deep phylogenetic relationships and extensive rate variation among palms and other commelinid monocots. New Phytol. 2016 Jan;209(2):855–70.
25. Fisher AE, Hasenstab KM, Bell HL, Blaine E, Ingram AL, Columbus JT. Evolutionary history of chloridoid grasses estimated from 122 nuclear loci. Mol Phylogenet Evol. 2016 Dec;105:1–14.
26. Welker CAD, Souza-Chies TT, Longhi-Wagner HM, Peichoto MC, McKain MR, Kellogg EA. Multilocus phylogeny and phylogenomics of Eriochrysis P. Beauv. (Poaceae-Andropogoneae): Taxonomic implications and evidence of interspecific hybridization. Mol Phylogenet Evol. 2016 Jun;99:155–67.
27. Washburn JD, Schnable JC, Davidse G, Pires JC. Phylogeny and photosynthesis of the grass tribe Paniceae. Am J Bot. 2015 Sep;102(9):1493–505.
28. Fjellheim S, Boden S, Trevaskis B. The role of seasonal flowering responses in adaptation of grasses to temperate climates. Front Plant Sci. 2014;5:431.
29. Hodkinson TR. Evolution and Taxonomy of the Grasses (Poaceae): A Model Family for the Study of Species-Rich Groups. Annu Plant Rev Online. 2018;1(1):255-293
30. Huang C-H, Sun R, Hu Y, Zeng L, Zhang N, Cai L, et al. Resolution of Brassicaceae Phylogeny Using Nuclear Genes Uncovers Nested Radiations and Supports Convergent Morphological Evolution. Mol Biol Evol. 2016 Feb;33(2):394–412.
31. Teh BT, Lim K, Yong CH, Ng CCY, Rao SR, Rajasegaran V, et al. The draft genome of tropical fruit durian (Durio zibethinus). Nat Genet. 2017 Nov;49(11):1633–41.
32. Zhao L, Li X, Zhang N, Zhang S-D, Yi T-S, Ma H, et al. Phylogenomic analyses of large-scale nuclear genes provide new insights into the evolutionary relationships within the rosids. Mol Phylogenet Evol. 2016 Dec;105:166–76.
33. Kreplak J, Madoui M-A, Cápal P, Novák P, Labadie K, Aubert G, et al. A reference genome for pea provides insight into legume genome evolution. Nat Genet. 2019 Sep;51(9):1411–22.
34. Cai L, Arnold BJ, Xi Z, Khost DE, Patel N, Hartmann CB, et al. Deeply Altered Genome Architecture in the Endoparasitic Flowering Plant Sapria himalayana Griff. (Rafflesiaceae). Curr Biol. 2021 Mar 8;31(5):1002-1011.e9.
35. Xu W, Zhang Q, Yuan W, Xu F, Muhammad Aslam M, Miao R, et al. The genome evolution and low-phosphorus adaptation in white lupin. Nat Commun. 2020 Feb 26;11(1):1069.
36. Cauz-Santos LA, Munhoz CF, Rodde N, Cauet S, Santos AA, Penha HA, et al. The Chloroplast Genome of Passiflora edulis (Passifloraceae) Assembled from Long Sequence Reads: Structural Organization and Phylogenomic Studies in Malpighiales. Front Plant Sci. 2017;8:334.
37. Fernandez-Pozo N, Menda N, Edwards JD, Saha S, Tecle IY, Strickler SR, et al. The Sol Genomics Network (SGN)—from genotype to phenotype to breeding. Nucleic Acids Research. 2015 Jan 28;43(D1):D1036–41.
38. Xu W-Q, Losh J, Chen C, Li P, Wang R-H, Zhao Y-P, et al. Comparative genomics of figworts (Scrophularia, Scrophulariaceae), with implications for the evolution of Scrophularia and Lamiales. Journal of Systematics and Evolution. 2019;57(1):55–65.
39. Wu F-Y, Tang C-Y, Guo Y-M, Bian Z-W, Fu J-Y, Lu G-H, et al. Transcriptome analysis explores genes related to shikonin biosynthesis in Lithospermeae plants and provides insights into Boraginales' evolutionary history. Sci Rep. 2017 Jun 30;7(1):4477.
40. Tank DC, Donoghue MJ. Phylogeny and Phylogenetic Nomenclature of the Campanulidae Based on an Expanded Sample of Genes and Taxa. sbot. 2010 Apr;35(2):425–41.

41. Walker JF, Yang Y, Feng T, Timoneda A, Mikenas J, Hutchison V, et al. From cacti to carnivores: Improved phylotranscriptomic sampling and hierarchical homology inference provide further insight into the evolution of Caryophyllales. American Journal of Botany. 2018;105(3):446–62.
42. Zhang C, Zhang T, Luebert F, Xiang Y, Huang C-H, Hu Y, et al. Asterid Phylogenomics/Phylotranscriptomics Uncover Morphological Evolutionary Histories and Support Phylogenetic Placement for Numerous Whole-Genome Duplications. Molecular Biology and Evolution. 2020 Nov 1;37(11):3188–210.
43. Worberg A, Quandt D, Barniske A-M, Löhne C, Hilu KW, Borsch T. Phylogeny of basal eudicots: Insights from non-coding and rapidly evolving DNA. Organisms Diversity & Evolution. 2007 Apr 12;7(1):55–77.
44. Ma Q, Sun T, Li S, Wen J, Zhu L, Yin T, et al. The Acer truncatum genome provides insights into nervonic acid biosynthesis. Plant J. 2020 Nov;104(3):662–78.
45. Wu H, Ma T, Kang M, Ai F, Zhang J, Dong G, et al. A high-quality Actinidia chinensis (kiwifruit) genome. Hortic Res. 2019 Oct 15;6:117.
46. Luo M-C, Gu YQ, Puiu D, Wang H, Twardziok SO, Deal KR, et al. Genome sequence of the progenitor of the wheat D genome Aegilops tauschii. Nature. 2017 Nov 23;551(7681):498–502.
47. Nguyen T-P, Mühlich C, Mohammadin S, van den Bergh E, Platts AE, Haas FB, et al. Genome Improvement and Genetic Map Construction for Aethionema arabicum, the First Divergent Branch in the Brassicaceae Family. G3 (Bethesda). 2019 Nov 5;9(11):3521–30.
48. Montgomery JS, Giacomini D, Waithaka B, Lanz C, Murphy BP, Campe R, et al. Draft Genomes of Amaranthus tuberculatus, Amaranthus hybridus, and Amaranthus palmeri. Genome Biol Evol. 2020 Nov 3;12(11):1988–93.
49. Amborella Genome Project. The Amborella genome and the evolution of flowering plants. Science. 2013 Dec 20;342(6165):1241089.
50. Ming R, VanBuren R, Wai CM, Tang H, Schatz MC, Bowers JE, et al. The pineapple genome and the evolution of CAM photosynthesis. Nat Genet. 2015 Dec;47(12):1435–42.
51. Xie J, Zhao H, Li K, Zhang R, Jiang Y, Wang M, et al. A chromosome-scale reference genome of Aquilegia oxysepala var. kansuensis. Hortic Res. 2020;7:113.
52. Rawat V, Abdelsamad A, Pietzenuk B, Seymour DK, Koenig D, Weigel D, et al. Improving the Annotation of Arabidopsis lyrata Using RNA-Seq Data. PLoS One. 2015;10(9):e0137391.
53. Cheng C-Y, Krishnakumar V, Chan AP, Thibaud-Nissen F, Schobel S, Town CD. Araport11: a complete reannotation of the Arabidopsis thaliana reference genome. Plant J. 2017 Feb;89(4):789–804.
54. Bertioli DJ, Jenkins J, Clevenger J, Dudchenko O, Gao D, Seijo G, et al. The genome sequence of segmental allotetraploid peanut Arachis hypogaea. Nat Genet. 2019 May;51(5):877–84.
55. Harkess A, Zhou J, Xu C, Bowers JE, Van der Hulst R, Ayyampalayam S, et al. The asparagus genome sheds light on the origin and evolution of a young Y chromosome. Nat Commun. 2017 Nov 2;8(1):1279.
56. Friis G, Vizueta J, Smith EG, Nelson DR, Khraiwesh B, Qudeimat E, et al. A high-quality genome assembly and annotation of the gray mangrove, Avicennia marina. G3 (Bethesda). 2021 Jan 18;11(1):jkaa025.
57. McGrath JM (Mitch), Funk A, Galewski P, Ou S, Townsend B, Davenport K, et al. A contiguous de novo genome assembly of sugar beet EL10 (Beta vulgaris L.) [Internet]. 2020 Oct [cited 2021 Sep 10] p. 2020.09.15.298315. Available from: https://www.biorxiv.org/content/10.1101/2020.09.15.298315v2
58. International Brachypodium Initiative. Genome sequencing and analysis of the model grass Brachypodium distachyon. Nature. 2010 Feb 11;463(7282):763–8.
59. Song X, Wei Y, Xiao D, Gong K, Sun P, Ren Y, et al. Brassica carinata genome characterization clarifies U's triangle model of evolution and polyploidy in Brassica. Plant Physiol. 2021 Feb 4;kiab048.

60. Rousseau-Gueutin M, Belser C, Da Silva C, Richard G, Istace B, Cruaud C, et al. Long-read assembly of the Brassica napus reference genome Darmor-bzh. Gigascience. 2020 Dec 15;9(12):giaa137.
61. Belser C, Istace B, Denis E, Dubarry M, Baurens F-C, Falentin C, et al. Chromosome-scale assemblies of plant genomes using nanopore long reads and optical maps. Nat Plants. 2018 Nov;4(11):879–87.
62. Zhao H, Wang S, Wang J, Chen C, Hao S, Chen L, et al. The chromosome-level genome assemblies of two rattans (Calamus simplicifolius and Daemonorops jenkinsiana). Gigascience. 2018 Sep 1;7(9).
63. Xia E, Tong W, Hou Y, An Y, Chen L, Wu Q, et al. The Reference Genome of Tea Plant and Resequencing of 81 Diverse Accessions Provide Insights into Its Genome Evolution and Adaptation. Mol Plant. 2020 Jul 6;13(7):1013–26.
64. Gao S, Wang B, Xie S, Xu X, Zhang J, Pei L, et al. A high-quality reference genome of wild Cannabis sativa. Hortic Res. 2020;7:73.
65. Slotte T, Hazzouri KM, Ågren JA, Koenig D, Maumus F, Guo Y-L, et al. The Capsella rubella genome and the genomic consequences of rapid mating system evolution. Nat Genet. 2013 Jul;45(7):831–5.
66. Kim S, Park M, Yeom S-I, Kim Y-M, Lee JM, Lee H-A, et al. Genome sequence of the hot pepper provides insights into the evolution of pungency in Capsicum species. Nat Genet. 2014 Mar;46(3):270–8.
67. Gan X, Hay A, Kwantes M, Haberer G, Hallab A, Ioio RD, et al. The Cardamine hirsuta genome offers insight into the evolution of morphological diversity. Nat Plants. 2016 Oct 31;2(11):16167.
68. Ming R, Hou S, Feng Y, Yu Q, Dionne-Laporte A, Saw JH, et al. The draft genome of the transgenic tropical fruit tree papaya (Carica papaya Linnaeus). Nature. 2008 Apr 24;452(7190):991–6.
69. Yang X, Wang Z, Zhang L, Hao G, Liu J, Yang Y. A chromosome-level reference genome of the hornbeam, Carpinus fangiana. Sci Data. 2020 Jan 21;7(1):24.
70. Lovell JT, Bentley NB, Bhattarai G, Jenkins JW, Sreedasyam A, Alarcon Y, et al. Four chromosome scale genomes and a pan-genome annotation to accelerate pecan tree breeding. Nat Commun. 2021 Jul 5;12(1):4125.
71. Yan Q, Wu F, Xu P, Sun Z, Li J, Gao L, et al. The elephant grass (Cenchrus purpureus) genome provides insights into anthocyanidin accumulation and fast growth. Mol Ecol Resour. 2021 Feb;21(2):526–42.
72. Nishiyama T, Sakayama H, de Vries J, Buschmann H, Saint-Marcoux D, Ullrich KK, et al. The Chara Genome: Secondary Complexity and Implications for Plant Terrestrialization. Cell. 2018 Jul 12;174(2):448-464.e24.
73. Jarvis DE, Ho YS, Lightfoot DJ, Schmöckel SM, Li B, Borm TJA, et al. The genome of Chenopodium quinoa. Nature. 2017 Feb 16;542(7641):307–12.
74. Merchant SS, Prochnik SE, Vallon O, Harris EH, Karpowicz SJ, Witman GB, et al. The Chlamydomonas genome reveals the evolution of key animal and plant functions. Science. 2007 Oct 12;318(5848):245–50.
75. Parween S, Nawaz K, Roy R, Pole AK, Venkata Suresh B, Misra G, et al. An advanced draft genome assembly of a desi type chickpea (Cicer arietinum L.). Sci Rep. 2015 Aug 11;5:12806.
76. Wu S, Wang X, Reddy U, Sun H, Bao K, Gao L, et al. Genome of “Charleston Gray”, the principal American watermelon cultivar, and genetic characterization of 1,365 accessions in the U.S. National Plant Germplasm System watermelon collection. Plant Biotechnol J. 2019 Dec;17(12):2246–58.
77. Wu GA, Prochnik S, Jenkins J, Salse J, Hellsten U, Murat F, et al. Sequencing of diverse mandarin, pummelo and orange genomes reveals complex history of admixture during citrus domestication. Nat Biotechnol. 2014 Jul;32(7):656–62.
78. Denoeud F, Carretero-Paulet L, Dereeper A, Droc G, Guyot R, Pietrella M, et al. The coffee genome provides insight into the convergent evolution of caffeine biosynthesis. Science. 2014 Sep 5;345(6201):1181–4.

79. Islam MS, Saito JA, Emdad EM, Ahmed B, Islam MM, Halim A, et al. Comparative genomics of two jute species and insight into fibre biogenesis. Nat Plants. 2017 Jan 30;3:16223.
80. Pavese V, Cavalet Giorsa E, Barchi L, Acquadro A, Torello Marinoni D, Portis E, et al. Whole-genome assembly of Corylus avellana cv'Tonda Gentile delle Langhe' using linked-reads (10X Genomics). G3 (Bethesda). 2021 May 8;jkab152.
81. Garcia-Mas J, Benjak A, Sanseverino W, Bourgeois M, Mir G, González VM, et al. The genome of melon (Cucumis melo L.). Proc Natl Acad Sci U S A. 2012 Jul 17;109(29):11872–7.
82. Li Q, Li H, Huang W, Xu Y, Zhou Q, Wang S, et al. A chromosome-scale genome assembly of cucumber (Cucumis sativus L.). Gigascience. 2019 Jun 1;8(6):giz072.
83. Iorizzo M, Ellison S, Senalik D, Zeng P, Satapoomin P, Huang J, et al. A high-quality carrot genome assembly provides new insights into carotenoid accumulation and asterid genome evolution. Nat Genet. 2016 Jun;48(6):657–66.
84. Chen Y, Ma T, Zhang L, Kang M, Zhang Z, Zheng Z, et al. Genomic analyses of a "living fossil": The endangered dove-tree. Mol Ecol Resour. 2020 May;20(3).
85. Abrouk M, Ahmed HI, Cubry P, Šimoníková D, Cauet S, Pailles Y, et al. Fonio millet genome unlocks African orphan crop diversity for agriculture in a changing climate. Nat Commun. 2020 Sep 8;11(1):4488.
86. Siadjeu C, Pucker B, Viehöver P, Albach DC, Weisshaar B. High Contiguity De Novo Genome Sequence Assembly of Trifoliate Yam (Dioscorea dumetorum) Using Long Read Sequencing. Genes (Basel). 2020 Mar 4;11(3):E274.
87. Singh R, Ong-Abdullah M, Low E-TL, Manaf MAA, Rosli R, Nookiah R, et al. Oil palm genome sequence reveals divergence of interfertile species in Old and New worlds. Nature. 2013 Aug 15;500(7462):335–9.
88. Carballo J, Santos BACM, Zappacosta D, Garbus I, Selva JP, Gallo CA, et al. A high-quality genome of Eragrostis curvula grass provides insights into Poaceae evolution and supports new strategies to enhance forage quality. Sci Rep. 2019 Jul 15;9:10250.
89. Laforest M, Martin SL, Bisaillon K, Soufiane B, Meloche S, Page E. A chromosome-scale draft sequence of the Canada fleabane genome. Pest Manag Sci. 2020 Jun;76(6):2158–69.
90. Hellsten U, Wright KM, Jenkins J, Shu S, Yuan Y, Wessler SR, et al. Fine-scale variation in meiotic recombination in Mimulus inferred from population shotgun sequencing. Proc Natl Acad Sci U S A. 2013 Nov 26;110(48):19478–82.
91. Myburg AA, Grattapaglia D, Tuskan GA, Hellsten U, Hayes RD, Grimwood J, et al. The genome of Eucalyptus grandis. Nature. 2014 Jun 19;510(7505):356–62.
92. Yang R, Jarvis DE, Chen H, Beilstein MA, Grimwood J, Jenkins J, et al. The Reference Genome of the Halophytic Plant Eutrema salsugineum. Front Plant Sci. 2013;4:46.
93. Li Y, Pi M, Gao Q, Liu Z, Kang C. Updated annotation of the wild strawberry Fragaria vesca V4 genome. Hortic Res. 2019;6:61.
94. Edger PP, Poorten TJ, VanBuren R, Hardigan MA, Colle M, McKain MR, et al. Origin and evolution of the octoploid strawberry genome. Nat Genet. 2019 Mar;51(3):541–7.
95. Valliyodan B, Cannon SB, Bayer PE, Shu S, Brown AV, Ren L, et al. Construction and comparison of three reference-quality genome assemblies for soybean. Plant J. 2019 Dec;100(5):1066–82.
96. Chen ZJ, Sreedasyam A, Ando A, Song Q, De Santiago LM, Hulse-Kemp AM, et al. Genomic diversifications of five Gossypium allopolyploid species and their impact on cotton improvement. Nat Genet. 2020 May;52(5):525–33.
97. Wang K, Wang Z, Li F, Ye W, Wang J, Song G, et al. The draft genome of a diploid cotton Gossypium raimondii. Nat Genet. 2012 Oct;44(10):1098–103.
98. Badouin H, Gouzy J, Grassa CJ, Murat F, Staton SE, Cottret L, et al. The sunflower genome provides insights into oil metabolism, flowering and Asterid evolution. Nature. 2017 Jun 1;546(7656):148–52.
99. Jayakodi M, Padmarasu S, Haberer G, Bonthala VS, Gundlach H, Monat C, et al. The barley pan-genome reveals the hidden legacy of mutation breeding. Nature. 2020 Dec;588(7837):284–9.

100. Nashima K, Shirasawa K, Ghelfi A, Hirakawa H, Isobe S, Suyama T, et al. Genome sequence of Hydrangea macrophylla and its application in analysis of the double flower phenotype. DNA Res. 2021 Jan 19;28(1):dsaa026.
101. Reyes-Chin-Wo S, Wang Z, Yang X, Kozik A, Arikit S, Song C, et al. Genome assembly with in vitro proximity ligation data and whole-genome triplication in lettuce. Nat Commun. 2017 Apr 12;8:14953.
102. Byrne SL, Nagy I, Pfeifer M, Armstead I, Swain S, Studer B, et al. A synteny-based draft genome sequence of the forage grass Lolium perenne. Plant J. 2015 Nov;84(4):816–26.
103. Pu X, Li Z, Tian Y, Gao R, Hao L, Hu Y, et al. The honeysuckle genome provides insight into the molecular mechanism of carotenoid metabolism underlying dynamic flower coloration. New Phytol. 2020 Aug;227(3):930–43.
104. Li H, Jiang F, Wu P, Wang K, Cao Y. A High-Quality Genome Sequence of Model Legume Lotus japonicus (MG-20) Provides Insights into the Evolution of Root Nodule Symbiosis. Genes (Basel). 2020 Apr 29;11(5):E483.
105. Hufnagel B, Marques A, Soriano A, Marquès L, Divol F, Doumas P, et al. High-quality genome sequence of white lupin provides insight into soil exploration and seed quality. Nat Commun. 2020 Jan 24;11(1):492.
106. Daccord N, Celton J-M, Linsmith G, Becker C, Choisne N, Schijlen E, et al. High-quality de novo assembly of the apple genome and methylome dynamics of early fruit development. Nat Genet. 2017 Jul;49(7):1099–106.
107. International Cassava Genetic Map Consortium (ICGMC). High-resolution linkage map and chromosome-scale genome assembly for cassava (Manihot esculenta Crantz) from 10 populations. G3 (Bethesda). 2014 Dec 11;5(1):133–44.
108. Bowman JL, Kohchi T, Yamato KT, Jenkins J, Shu S, Ishizaki K, et al. Insights into Land Plant Evolution Garnered from the Marchantia polymorpha Genome. Cell. 2017 Oct 5;171(2):287-304.e15.
109. Young ND, Debellé F, Oldroyd GED, Geurts R, Cannon SB, Udvardi MK, et al. The Medicago genome provides insight into the evolution of rhizobial symbioses. Nature. 2011 Nov 16;480(7378):520–4.
110. Worden AZ, Lee J-H, Mock T, Rouzé P, Simmons MP, Aerts AL, et al. Green evolution and dynamic adaptations revealed by genomes of the marine picoeukaryotes Micromonas. Science. 2009 Apr 10;324(5924):268–72.
111. Mitros T, Session AM, James BT, Wu GA, Belaffif MB, Clark LV, et al. Genome biology of the paleotetraploid perennial biomass crop Miscanthus. Nat Commun. 2020 Oct 28;11(1):5442.
112. D'Hont A, Denoeud F, Aury J-M, Baurens F-C, Carreel F, Garsmeur O, et al. The banana (Musa acuminata) genome and the evolution of monocotyledonous plants. Nature. 2012 Aug 9;488(7410):213–7.
113. Li H, Yang X, Zhang Y, Gao Z, Liang Y, Chen J, et al. Nelumbo genome database, an integrative resource for gene expression and variants of Nelumbo nucifera. Sci Data. 2021 Jan 29;8(1):38.
114. Edwards KD, Fernandez-Pozo N, Drake-Stowe K, Humphry M, Evans AD, Bombarely A, et al. A reference genome for Nicotiana tabacum enables map-based cloning of homeologous loci implicated in nitrogen utilization efficiency. BMC Genomics. 2017 Jun 19;18(1):448.
115. Cruz F, Julca I, Gómez-Garrido J, Loska D, Marcet-Houben M, Cano E, et al. Genome sequence of the olive tree, Olea europaea. Gigascience. 2016 Jun 27;5:29.
116. VanBuren R, Wai CM, Keilwagen J, Pardo J. A chromosome-scale assembly of the model desiccation tolerant grass Oropetium thomaeum. Plant Direct. 2018 Nov;2(11):e00096.
117. Yu H, Lin T, Meng X, Du H, Zhang J, Liu G, et al. A route to de novo domestication of wild allotetraploid rice. Cell. 2021 Mar 4;184(5):1156-1170.e14.
118. Jacquemin J, Bhatia D, Singh K, Wing RA. The International Oryza Map Alignment Project: development of a genus-wide comparative genomics platform to help solve the 9 billion-people question. Curr Opin Plant Biol. 2013 May;16(2):147–56.

119. Jain R, Jenkins J, Shu S, Chern M, Martin JA, Copetti D, et al. Genome sequence of the model rice variety KitaakeX. BMC Genomics. 2019 Nov 27;20(1):905.
120. Yu J, Hu S, Wang J, Wong GK-S, Li S, Liu B, et al. A draft sequence of the rice genome (Oryza sativa L. ssp. indica). Science. 2002 Apr 5;296(5565):79–92.
121. International Rice Genome Sequencing Project. The map-based sequence of the rice genome. Nature. 2005 Aug 11;436(7052):793–800.
122. Lovell JT, Jenkins J, Lowry DB, Mamidi S, Sreedasyam A, Weng X, et al. The genomic landscape of molecular responses to natural drought stress in Panicum hallii. Nat Commun. 2018 Dec 6;9(1):5213.
123. Guo L, Winzer T, Yang X, Li Y, Ning Z, He Z, et al. The opium poppy genome and morphinan production. Science. 2018 Oct 19;362(6412):343–7.
124. Bombarely A, Moser M, Amrad A, Bao M, Bapaume L, Barry CS, et al. Insight into the evolution of the Solanaceae from the parental genomes of Petunia hybrida. Nat Plants. 2016 May 27;2(6):16074.
125. Phytozome info: P.vulgaris v2.1 [Internet]. [cited 2021 Sep 10]. Available from: https://phytozome-next.jgi.doe.gov/info/Pvulgaris_v2_1
126. Zhao H, Gao Z, Wang L, Wang J, Wang S, Fei B, et al. Chromosome-level reference genome and alternative splicing atlas of moso bamboo (Phyllostachys edulis). Gigascience. 2018 Oct 1;7(10).
127. Rensing SA, Lang D, Zimmer AD, Terry A, Salamov A, Shapiro H, et al. The Physcomitrella genome reveals evolutionary insights into the conquest of land by plants. Science. 2008 Jan 4;319(5859):64–9.
128. Tuskan GA, Difazio S, Jansson S, Bohlmann J, Grigoriev I, Hellsten U, et al. The genome of black cottonwood, Populus trichocarpa (Torr. & Gray). Science. 2006 Sep 15;313(5793):1596–604.
129. Li L, Wang S, Wang H, Sahu SK, Marin B, Li H, et al. The genome of Prasinoderma coloniale unveils the existence of a third phylum within green plants. Nat Ecol Evol. 2020 Sep;4(9):1220–31.
130. International Peach Genome Initiative, Verde I, Abbott AG, Scalabrin S, Jung S, Shu S, et al. The high-quality draft genome of peach (Prunus persica) identifies unique patterns of genetic diversity, domestication and genome evolution. Nat Genet. 2013 May;45(5):487–94.
131. Qin G, Xu C, Ming R, Tang H, Guyot R, Kramer EM, et al. The pomegranate (Punica granatum L.) genome and the genomics of punicalagin biosynthesis. Plant J. 2017 Sep;91(6):1108–28.
132. Sork VL, Fitz-Gibbon ST, Puiu D, Crepeau M, Gugger PF, Sherman R, et al. First Draft Assembly and Annotation of the Genome of a California Endemic Oak Quercus lobata Née (Fagaceae). G3 (Bethesda). 2016 Nov 8;6(11):3485–95.
133. Yang F-S, Nie S, Liu H, Shi T-L, Tian X-C, Zhou S-S, et al. Chromosome-level genome assembly of a parent species of widely cultivated azaleas. Nat Commun. 2020 Oct 19;11(1):5269.
134. Raymond O, Gouzy J, Just J, Badouin H, Verdenaud M, Lemainque A, et al. The Rosa genome provides new insights into the domestication of modern roses. Nat Genet. 2018 Jun;50(6):772–7.
135. Zhang J, Zhang X, Tang H, Zhang Q, Hua X, Ma X, et al. Allele-defined genome of the autopolyploid sugarcane Saccharum spontaneum L. Nat Genet. 2018 Nov;50(11):1565–73.
136. Chen J-H, Huang Y, Brachi B, Yun Q-Z, Zhang W, Lu W, et al. Genome-wide analysis of Cushion willow provides insights into alpine plant divergence in a biodiversity hotspot. Nat Commun. 2019 Nov 19;10(1):5230.
137. Zheng X, Chen D, Chen B, Liang L, Huang Z, Fan W, et al. Insights into salvianolic acid B biosynthesis from chromosome-scale assembly of the Salvia bowleyana genome. J Integr Plant Biol. 2021 Jul;63(7):1309–23.
138. Dassanayake M, Oh D-H, Haas JS, Hernandez A, Hong H, Ali S, et al. The genome of the extremophile crucifer Thellungiella parvula. Nat Genet. 2011 Aug 7;43(9):913–8.

139. Li G, Wang L, Yang J, He H, Jin H, Li X, et al. A high-quality genome assembly highlights rye genomic characteristics and agronomically important genes. Nat Genet. 2021 Apr;53(4):574–84.
140. Fu A, Wang Q, Mu J, Ma L, Wen C, Zhao X, et al. Combined genomic, transcriptomic, and metabolomic analyses provide insights into chayote (Sechium edule) evolution and fruit development. Hortic Res. 2021 Jan 31;8(1):35.
141. Banks JA, Nishiyama T, Hasebe M, Bowman JL, Gribskov M, dePamphilis C, et al. The Selaginella genome identifies genetic changes associated with the evolution of vascular plants. Science. 2011 May 20;332(6032):960–3.
142. Zheng J, Meinhardt LW, Goenaga R, Zhang D, Yin Y. The chromosome-level genome of dragon fruit reveals whole-genome duplication and chromosomal co-localization of betacyanin biosynthetic genes. Hortic Res. 2021 Mar 10;8(1):63.
143. Scott AD, Zimin AV, Puiu D, Workman R, Britton M, Zaman S, et al. A Reference Genome Sequence for Giant Sequoia. G3 (Bethesda). 2020 Nov 5;10(11):3907–19.
144. Zhang G, Liu X, Quan Z, Cheng S, Xu X, Pan S, et al. Genome sequence of foxtail millet (Setaria italica) provides insights into grass evolution and biofuel potential. Nat Biotechnol. 2012 May 13;30(6):549–54.
145. Mamidi S, Healey A, Huang P, Grimwood J, Jenkins J, Barry K, et al. A genome resource for green millet Setaria viridis enables discovery of agronomically valuable loci. Nat Biotechnol. 2020 Oct;38(10):1203–10.
146. Sturtevant D, Lu S, Zhou Z-W, Shen Y, Wang S, Song J-M, et al. The genome of jojoba (Simmondsia chinensis): A taxonomically isolated species that directs wax ester accumulation in its seeds. Sci Adv. 2020 Mar;6(11):eaay3240.
147. Tomato Genome Consortium. The tomato genome sequence provides insights into fleshy fruit evolution. Nature. 2012 May 30;485(7400):635–41.
148. Bolger A, Scossa F, Bolger ME, Lanz C, Maumus F, Tohge T, et al. The genome of the stress-tolerant wild tomato species Solanum pennellii. Nat Genet. 2014 Sep;46(9):1034–8.
149. Potato Genome Sequencing Consortium, Xu X, Pan S, Cheng S, Zhang B, Mu D, et al. Genome sequence and analysis of the tuber crop potato. Nature. 2011 Jul 10;475(7355):189–95.
150. Paterson AH, Bowers JE, Bruggmann R, Dubchak I, Grimwood J, Gundlach H, et al. The Sorghum bicolor genome and the diversification of grasses. Nature. 2009 Jan 29;457(7229):551–6.
151. Wang W, Haberer G, Gundlach H, Gläßer C, Nussbaumer T, Luo MC, et al. The Spirodela polyrhiza genome reveals insights into its neotenous reduction fast growth and aquatic lifestyle. Nat Commun. 2014;5:3311.
152. Yoshida S, Kim S, Wafula EK, Tanskanen J, Kim Y-M, Honaas L, et al. Genome Sequence of Striga asiatica Provides Insight into the Evolution of Plant Parasitism. Curr Biol. 2019 Sep 23;29(18):3041-3052.e4.
153. Cheng S, van den Bergh E, Zeng P, Zhong X, Xu J, Liu X, et al. The Tarenaya hassleriana genome provides insight into reproductive trait and genome evolution of crucifers. Plant Cell. 2013 Aug;25(8):2813–30.
154. Motamayor JC, Mockaitis K, Schmutz J, Haiminen N, Livingstone D, Cornejo O, et al. The genome sequence of the most widely cultivated cacao type and its use to identify candidate genes regulating pod color. Genome Biol. 2013 Jun 3;14(6):r53.
155. De Vega JJ, Ayling S, Hegarty M, Kudrna D, Goicoechea JL, Ergon Å, et al. Red clover (Trifolium pratense L.) draft genome provides a platform for trait improvement. Sci Rep. 2015 Nov 30;5:17394.
156. Tu L, Su P, Zhang Z, Gao L, Wang J, Hu T, et al. Genome of Tripterygium wilfordii and identification of cytochrome P450 involved in triptolide biosynthesis. Nat Commun. 2020 Feb 20;11(1):971.

157. International Wheat Genome Sequencing Consortium (IWGSC). A chromosome-based draft sequence of the hexaploid bread wheat (Triticum aestivum) genome. Science. 2014 Jul 18;345(6194):1251788.
158. Avni R, Nave M, Barad O, Baruch K, Twardziok SO, Gundlach H, et al. Wild emmer genome architecture and diversity elucidate wheat evolution and domestication. Science. 2017 Jul 7;357(6346):93–7.
159. Maccaferri M, Harris NS, Twardziok SO, Pasam RK, Gundlach H, Spannagl M, et al. Durum wheat genome highlights past domestication signatures and future improvement targets. Nat Genet. 2019 May;51(5):885–95.
160. Strijk JS, Hinsinger DD, Zhang F, Cao K. Trochodendron aralioides, the first chromosome-level draft genome in Trochodendrales and a valuable resource for basal eudicot research. Gigascience. 2019 Nov 1;8(11):giz136.
161. Lan T, Renner T, Ibarra-Laclette E, Farr KM, Chang T-H, Cervantes-Pérez SA, et al. Long-read sequencing uncovers the adaptive topography of a carnivorous plant genome. Proc Natl Acad Sci U S A. 2017 May 30;114(22):E4435–41.
162. Diaz-Garcia L, Garcia-Ortega LF, González-Rodríguez M, Delaye L, Iorizzo M, Zalapa J. Chromosome-Level Genome Assembly of the American Cranberry (Vaccinium macrocarpon Ait.) and Its Wild Relative Vaccinium microcarpum. Front Plant Sci. 2021;12:633310.
163. Hasing T, Tang H, Brym M, Khazi F, Huang T, Chambers AH. A phased Vanilla planifolia genome enables genetic improvement of flavour and production. Nat Food. 2020 Dec;1(12):811–9.
164. Pootakham W, Nawae W, Naktang C, Sonthirod C, Yoocha T, Kongkachana W, et al. A chromosome-scale assembly of the black gram (Vigna mungo) genome. Mol Ecol Resour. 2021 Jan;21(1):238–50.
165. Jaillon O, Aury J-M, Noel B, Policriti A, Clepet C, Casagrande A, et al. The grapevine genome sequence suggests ancestral hexaploidization in major angiosperm phyla. Nature. 2007 Sep 27;449(7161):463–7.
166. Woodhouse MR, Cannon EK, Portwood JL, Harper LC, Gardiner JM, Schaeffer ML, et al. A pan-genomic approach to genome databases using maize as a model system. BMC Plant Biology. 2021 Aug 20;21(1):385.
167. Schnable PS, Ware D, Fulton RS, Stein JC, Wei F, Pasternak S, et al. The B73 maize genome: complexity, diversity, and dynamics. Science. 2009 Nov 20;326(5956):1112–5.
168. Olsen JL, Rouzé P, Verhelst B, Lin Y-C, Bayer T, Collen J, et al. The genome of the seagrass Zostera marina reveals angiosperm adaptation to the sea. Nature. 2016 Feb;530(7590):331–5.
169. Tanaka H, Hirakawa H, Kosugi S, Nakayama S, Ono A, Watanabe A, et al. Sequencing and comparative analyses of the genomes of zoysiagrasses. DNA Res. 2016 Apr;23(2):171–80.
